# Dietary Patterns and an Adverse Mental Health Indicator Among School-Attending Brazilian Adolescents: Analyses of the 2015 and 2019 *PeNSE* Surveys

**DOI:** 10.3390/nu18172781

**Published:** 2026-08-25

**Authors:** Lídia Sterza Vasconcellos, Woska Pires da Costa, Aline Bergamini Effgen Sena, Wesley Rocha Grippa, Luiz Claudio Barreto Silva Neto, Vitor Engrácia Valenti, Weverton Pereira de Medeiros, Luana da Silva Baptista Arpini, Luiz Carlos de Abreu

**Affiliations:** 1 Postgraduate Program in Nutrition and Health, Universidade Federal do Espírito Santo (UFES), Marechal Campos Avenue, No. 1468, Maruípe, Vitória 29040-090, ES, Brazil; lidiasterza.nutri@outlook.com (L.S.V.); alineffgen@gmail.com (A.B.E.S.); wevertonmedeiros74@gmail.com (W.P.d.M.); luanaarpini@hotmail.com (L.d.S.B.A.); 2 Medical School, Universidade de São Paulo (USP), Dr. Arnaldo Avenue, No. 455, Cerqueira César Neighborhood, Central Institute of Clinical Hospital Building, Block II, 2nd Floor, P.O. Box 8091, São Paulo 01246-903, SP, Brazil; woskapc@gmail.com; 3 Research Department, Instituto Federal Goiano—Campus Morrinhos, BR-153 Highway, Km 633, Rural Area, P.O. Box 92, Morrinhos 75658-899, GO, Brazil; 4 Department of Applied Mathematics, Northern Espírito Santo University Center, Universidade Federal do Espírito Santo (UFES), BR-101 North Highway, Km 60, Litorâneo Neighborhood, São Mateus 29932-540, ES, Brazil; wgripa@gmail.com; 5 Postgraduate Program in Public Health, Center for Health Sciences, Universidade Federal do Espírito Santo (UFES), Marechal Campos Avenue, No. 1468, Maruípe, Vitória 29047-105, ES, Brazil; 6 School of Philosophy and Sciences—Systematic Reviews Center for Cardiovascular and Metabolic Health, Universidade Estadual Paulista (UNESP), Hygino Muzzi Filho Avenue, No. 737, Mirante Neighborhood, Marília 17525-900, SP, Brazil; vitor.valenti@unesp.br; 7 Espírito Santo State Health Secretariat, Enseada Plaza Building, Engenheiro Guilherme José Monjardim Varejão Street, No. 225, Enseada do Suá Neighborhood, Vitória 29050-260, ES, Brazil; 8 Capixaba Institute of Teaching, Research and Innovation, State Health Secretariat of Espírito Santo, Duque de Caxias Street, No. 267, City Center, Vitória 29010-120, ES, Brazil; 9 Department of Rehabilitation Sciences, Florida Gulf Coast University (FGCU), FGCU Boulevard South, Marieb Hall Building, No. 10501, Fort Myers, FL 33965-6565, USA; 10 School of Medicine, Faculty of Education and Health Sciences, University of Limerick (UL), Castletroy, V94 T9PX Co. Limerick, Ireland

**Keywords:** adolescent health, dietary patterns, mental health, ultra-processed foods, healthy diet, school health, students

## Abstract

Background/Objectives: Dietary patterns may be associated with adolescent mental health, but nationwide Brazilian evidence based on empirically derived patterns across survey editions remains limited. This study identified dietary patterns and examined their associations with a survey-specific adverse mental health indicator among school-attending adolescents aged 13–17 years in the 2015 and 2019 National Survey of School Health (*PeNSE*). Methods: The two independent cross-sectional surveys were analyzed separately, with the 2015 analysis restricted to Sample 2. Two dietary patterns were derived in each edition using principal component analysis. The primary outcome was membership in the highest category defined from the weighted distribution of a non-diagnostic, survey-specific composite score of adverse mental health-related indicators. Survey-weighted modified Poisson regression estimated adjusted prevalence ratios (PRs); continuous, ordinal, between-survey heterogeneity, alternative-outcome, weighted-PCA, and E-value analyses assessed robustness. Results: Both surveys yielded ultra-processed/unhealthy and healthy/traditional patterns. Compared with the highest adherence quartile (Q4), the lowest quartile (Q1) of the ultra-processed/unhealthy pattern was associated with a lower prevalence of the adverse indicator in 2015 (adjusted PR = 0.85, 95% CI: 0.73–0.98) and 2019 (adjusted PR = 0.81, 95% CI: 0.76–0.86). Conversely, Q1 of the healthy/traditional pattern was associated with a higher prevalence in 2015 (adjusted PR = 1.75, 95% CI: 1.45–2.10) and 2019 (adjusted PR = 1.34, 95% CI: 1.26–1.42). No between-survey heterogeneity was detected for the ultra-processed/unhealthy pattern, whereas the healthy/traditional estimates differed in magnitude between surveys; sensitivity analyses were directionally consistent. Conclusions: Greater ultra-processed/unhealthy adherence and lower healthy/traditional adherence were associated with a higher prevalence of the survey-specific adverse mental health indicator. Because the surveys were cross-sectional and used edition-specific measures, these findings do not establish temporality, causality, or temporal change in invariant constructs.

## 1. Introduction

Adolescence is a sensitive developmental period marked by rapid biological, psychological, and social changes that shape health trajectories extending into adulthood. Mental disorders account for a substantial proportion of disability worldwide, and many first emerge during childhood, adolescence, or early adulthood, with potential consequences for educational attainment, social relationships, health behaviors, and later quality of life [1,2,3]. Early identification of modifiable correlates of adverse mental health is therefore an important public health priority. Because schools reach large and socially diverse groups of young people, they offer a strategic setting for population surveillance, prevention, and health-promotion initiatives, although the effectiveness of school-based interventions may vary according to their design, intensity, and context [4].

Diet is another central dimension of adolescent health. Contemporary food environments combine traditional and health-promoting foods with widespread access to ultra-processed products. Under the NOVA framework, ultra-processed foods are industrial formulations composed largely of modified food substances and additives designed to enhance convenience, palatability, and commercial appeal [5]. In Brazil, dietary change is heterogeneous and socially patterned, reflecting a complex nutritional transition rather than a uniform replacement of traditional foods. Persistent exposure to ultra-processed products coexists with variable consumption of beans, fruits, vegetables, sweets, and soft drinks across population groups and contexts [6,7]. Individual preferences, family routines, school environments, socioeconomic conditions, food accessibility, and broader commercial influences shape these coexisting practices. A systematic review of Brazilian adolescents identified screen time, private-school attendance, higher body mass index, and female sex as recurrent correlates of ultra-processed food consumption [8]. Because foods are consumed in combination, dietary-pattern analysis can represent correlated eating behaviors more comprehensively than single-food or single-nutrient approaches [9]. However, empirically derived patterns depend on the available dietary markers, population characteristics, preprocessing procedures, component-retention criteria, and substantive labeling and therefore require transparent, population-specific interpretation [10].

Several interconnected biological, behavioral, and social pathways may help explain associations between dietary patterns and mental health. Diet quality may relate to nutrient adequacy, systemic inflammation, oxidative stress, neuroplasticity, hypothalamic–pituitary–adrenal axis regulation, tryptophan metabolism, and microbiota–gut–brain signaling [11,12]. In a regional study of older Brazilian adolescents, greater adherence to a prudent pattern rich in fresh and minimally processed foods was associated with lower interleukin-6 concentrations, whereas greater adiposity was associated with higher high-sensitivity C-reactive protein concentrations [13]. These findings support biological plausibility but do not establish that inflammation mediates diet–mental health associations. Dietary behaviors also cluster with sleep, physical activity, family routines, school experiences, and socioeconomic conditions, while emotional distress may influence appetite, meal timing, food preferences, and stress-related eating. Consequently, cross-sectional associations are compatible with bidirectionality and shared determinants and should not be interpreted as evidence that dietary behavior alone causes mental health problems [14].

Evidence on the diet–mental health relationship should be interpreted in light of population characteristics, exposure and outcome definitions, and study design. A meta-analysis of 17 observational studies found that greater ultra-processed food consumption was associated with depressive and anxiety symptoms and, in two prospective studies, with subsequent depression; however, the evidence base was predominantly cross-sectional and not specific to adolescents [15]. In an adolescent-focused synthesis of six intervention trials and 13 prospective cohorts, healthier dietary patterns were often associated with fewer depressive symptoms, although findings were heterogeneous and constrained by differences in dietary and mental health assessments, small samples, risk of bias, and sensitivity to sex and socioeconomic adjustment [16]. A more recent review of children and adolescents similarly found that most studies linked higher ultra-processed food intake to adverse mental health outcomes, while emphasizing variation by country, sex, exposure definition, and outcome measurement, as well as the scarcity of longitudinal and interventional evidence [17].

In the Brazilian context, ultra-processed food consumption has been associated with internalizing symptoms, common mental disorders, and less favorable mental health-related indicators among adolescents [18,19,20]. The closest national comparison showed that consumption of a greater number of ultra-processed food types during the previous 24 h was associated with more frequent mental health symptoms in both sexes; nevertheless, its count-based dietary exposure and five-symptom score differ from the seven-day, empirically derived dietary patterns and survey-specific adverse mental health indicators examined in the present study [20]. Comparable associations have also been observed among Brazilian education workers, in whom more frequent consumption of ultra-processed foods, soft drinks, sweets, and fried snacks was associated with higher depression, anxiety, and stress scores, whereas fruit and vegetable consumption was associated with more favorable mental health and quality-of-life indicators [21].

The National Survey of School Health (*Pesquisa Nacional de Saúde do Escolar*—*PeNSE*) provides a valuable platform for examining dietary and mental health indicators in large samples of school-attending adolescents. *PeNSE* is a nationwide school-based survey of students enrolled in public and private schools; in the 2015 and 2019 editions, participants completed structured, self-administered electronic questionnaires during school hours [22,23]. Analyses using *PeNSE* data have identified heterogeneous dietary and nutritional profiles embedded in socioeconomic, family, school, behavioral, and psychosocial contexts [24]. Analyses of comparable subsamples from 2009 to 2019 have also shown that the occurrence and socioeconomic distribution of multiple health-risk behaviors varied across survey periods, highlighting the importance of considering shared social and contextual determinants when examining diet and mental health [25]. However, differences in dietary markers, mental health items, sampling domains, and questionnaire structure limit direct measurement equivalence between the 2015 and 2019 editions [26]. Consequently, evidence formally comparing the magnitude of associations between empirically derived dietary patterns and multidimensional, survey-specific mental health indicators across these independent nationwide surveys remains limited.

Therefore, this study aimed to analyze dietary patterns and their associations with an adverse mental health indicator among school-attending Brazilian adolescents aged 13–17 years using data from the 2015 and 2019 editions of *PeNSE*. By analyzing each edition separately and formally comparing the magnitude of the resulting associations, this study may strengthen the evidence base for adolescent health surveillance and for integrated school and public health strategies addressing nutrition and mental well-being.

## 2. Methods

### 2.1. Study Design and Data Source

This observational, analytical study was a secondary analysis of data from two independent cross-sectional editions of *PeNSE*. Because exposures and outcomes were assessed within the same survey period, temporal sequence could not be established, and causal inferences were not supported [27,28,29]. As different participants were sampled in 2015 and 2019, the study did not constitute a longitudinal follow-up of the same individuals.

Data were obtained from the 2015 and 2019 editions of *PeNSE*. Each edition used a stratified, multistage cluster sampling design, although the sampling frames, target populations, and analytical domains differed across surveys. Sampling weights provided by *IBGE* accounted for unequal selection probabilities and nonresponse. *PeNSE* 2015 [30] comprised two independent sampling plans. The present study was restricted to Sample 2, which was specifically designed to represent school-attending adolescents aged 13–17 years and was independent of Sample 1, which targeted ninth-grade students [22,30]. The 2019 analysis included participants aged 13–17 years from the corresponding nationwide school-based survey sample [23,31]. Because the two editions represented independent samples and differed in the available dietary and mental health indicators, all variables and analyses were handled separately for each survey [26].

### 2.2. Study Population and Sample Definition

The target population comprised school-attending Brazilian adolescents aged 13–17 years enrolled in public or private schools. For *PeNSE* 2015, the study was restricted to Sample 2, an independent sampling plan specifically designed to represent students in this age range. Sample 1, which targeted ninth-grade students under a distinct sampling design, was not included. For *PeNSE* 2019 [31], the study included participants aged 13–17 years from the corresponding nationwide school-based survey sample.

Participants were eligible when their reported age was 13–17 years. The principal component analysis (PCA) estimation required complete data for all survey-specific dietary markers; the weighted PCA additionally required a valid final sampling weight. Crude association models required a valid primary mental health outcome and valid sampling-design variables, whereas adjusted models additionally required complete data for all prespecified covariates. Participants with unreported sex in the 2019 survey were therefore retained in PCA estimation and crude models but excluded from adjusted models. No missing-data imputation was performed.

### 2.3. Dietary Markers

Dietary intake was assessed as the number of days during the seven days preceding the survey on which each food or food group was consumed. The available dietary markers differed between the two editions. The *PeNSE* 2015 analysis included beans, vegetables or leafy greens, fresh fruit, soft drinks, sweets, ultra-processed foods, savory snacks, and snacks. The *PeNSE* 2019 analysis included beans, vegetables or leafy greens, fresh fruit, soft drinks, sweets, and fast food. The differences in the available markers reflect changes in the *PeNSE* questionnaires between editions [22,23,26]. Variables were coded so that higher values represented increasing frequency of consumption.

### 2.4. Dietary-Pattern Derivation

Dietary patterns were derived separately for each survey among age-eligible participants with complete data for all survey-specific dietary markers. The primary PCA used an unweighted Pearson correlation matrix. A supplementary PCA used a Pearson correlation matrix weighted by the final sampling weights; strata and primary sampling units were not used to derive this matrix. A two-component solution was prespecified to support comparable substantive interpretation across editions, based on prior exploratory analyses and the expectation of coexisting ultra-processed/unhealthy and healthy/traditional profiles among Brazilian adolescents. Retention was then evaluated empirically within each survey using the observed eigenvalues, scree-plot inspection [32], the Kaiser criterion [33], Horn parallel analysis [34] based on 5000 random correlation matrices with the same sample size and number of markers, explained variance, and interpretability of the rotated loading structure. The use and interpretation of PCA for empirically derived dietary patterns were additionally informed by methodological guidance specific to nutritional epidemiology [10]. Components were retained only when the overall diagnostic evidence supported a parsimonious and nutritionally coherent solution; conceptual comparability was not treated as evidence of empirical measurement equivalence.

For the primary unweighted PCA, matrix suitability was evaluated using the global Kaiser–Meyer–Olkin (KMO) statistic [35], marker-specific measures of sampling adequacy, the determinant and eigenvalues of the correlation matrix, and Bartlett’s test of sphericity [36]. The weighted PCA was assessed using the same descriptive matrix diagnostics, but no conventional inferential Bartlett test was applied. For both solutions, communalities, loading strength and cross-loadings, explained variance, reproduced-correlation residuals, Varimax convergence, score-coefficient estimability, and structural alignment were examined. Prespecified rules distinguished critical mathematical failures from nonfatal warnings; the latter—including communalities below 0.30, explained variance below 50%, and absolute off-diagonal residuals above 0.05—were documented but did not automatically trigger marker removal. Both PCA families used orthogonal Varimax rotation with Kaiser normalization [37].

Components were labeled according to the dietary markers with the highest positive loadings and were designated the ultra-processed/unhealthy and healthy/traditional dietary patterns. Component order and sign were standardized so that higher scores represented greater adherence to the named pattern. Individual scores were calculated by the regression scoring method. Primary unweighted scores were standardized using the mean and sample standard deviation (*SD*) (denominator *n* − 1) of the respective PCA sample. Supplementary weighted scores were standardized using the sampling-weighted mean and population-weighted *SD*. Adherence quartiles were defined by empirical cumulative distributions without interpolation, preserving ties: unweighted distributions for the primary analyses and final-weighted distributions for the weighted-PCA robustness analyses. Q1 represented the lowest and Q4 the highest adherence, with Q4 used as the reference category. These quartiles represent relative positions within each survey-specific score distribution; therefore, Q4 denotes the highest relative adherence group in that edition and not a common absolute adherence threshold across 2015 and 2019.

### 2.5. Survey-Specific Composite Scores of Adverse Mental Health-Related Indicators and Adverse-Score Outcome

Survey-specific composite scores of adverse mental health-related indicators (non-diagnostic composite indicators) were constructed because the available indicators differed between *PeNSE* editions. The 2015 score included items on parental understanding, peer support, peer mistreatment, having experienced bullying, loneliness, sleep loss due to worry, and the number of close friends. The 2019 score included the number of close friends and the frequency of worry, sadness, perceived lack of care, irritability or nervousness, and feeling that life was not worth living. These scores were constructed to summarize the relative burden of mental health–related experiences and symptoms across each survey edition. They were intended for epidemiological ranking and population surveillance and should not be interpreted as validated diagnostic scales or measures of specific mental disorders.

Item responses were recoded, including reverse-coding of positively framed or protective items as necessary, so that higher item and total scores consistently represented a greater burden of the adverse mental health-related experiences and symptoms captured by each survey-specific adverse mental health-related indicator score, rather than poorer mental health itself. The recoded item scores were summed without differential weighting; a total score was calculated only for participants with no missing responses to all survey-specific items, yielding theoretical ranges of 7–31 in 2015 and 5–28 in 2019. Survey-specific cut points corresponding to the weighted 25th, 50th, and 75th cumulative-distribution positions were estimated among age-eligible participants with a valid total score and final sampling weight, without interpolation and preserving ties. The four ordered categories were ≤11, 12–14, 15–17, and ≥18 in 2015 and ≤11, 12–15, 16–18, and ≥19 in 2019. The primary survey-specific adverse-score indicator was membership in the highest category. Because no clinically validated thresholds exist for these survey-specific adverse mental health-related indicator scores, the outcome identifies the relatively most adverse segment of each adverse mental health-related indicator score distribution. It should not be interpreted as a direct measure of mental health or as the prevalence of a clinically defined mental disorder. Ties in the discrete scores meant that the categories did not necessarily contain exactly 25% of participants. The source-variable names, concise questionnaire wording, recall periods, response-to-score mappings, and survey-specific category cut points are reported in Table S1.

For the sensitivity analysis, the survey-specific adverse-score indicator was alternatively defined as membership in either of the two upper-weighted score categories rather than the highest category alone: scores ≥ 15 vs. ≤14 in 2015, and scores ≥ 16 vs. ≤15 in 2019. Because item composition, recall periods, and possible score ranges differed across survey editions, the raw total scores were neither pooled nor interpreted as metrically equivalent measures. All analyses therefore used survey-specific outcome definitions, and between-survey comparisons refer to differences in the magnitude of survey-specific associations rather than temporal changes in a metrically invariant measure of mental health.

Internal consistency was assessed separately for each survey among PCA-eligible participants with complete mental health item data and valid survey-design information, corresponding to the primary crude analytical domain. Conventional unweighted Cronbach’s *α* was the primary reliability estimate, and a sampling-weighted coefficient based on the weighted item covariance matrix was calculated as a sensitivity analysis [38]. Cronbach’s *α* was interpreted only as a measure of inter-item consistency.

Taken together, these properties support interpretation of the scores as survey-specific adverse mental health-related indicators of relative psychosocial and emotional burden rather than psychometric scales. Their content covers internalizing experiences, perceived social connectedness or support, peer adversity, and, in 2015, sleep disruption related to worry. This content relevance is appropriate for population surveillance, but it does not establish factorial or construct validity, criterion validity, diagnostic accuracy, or measurement invariance. The lower 2015 alpha indicates that its items form a broader, less homogeneous summary than the 2019 set, implying greater measurement error. Accordingly, the analyses used survey-specific relative thresholds, examined an alternative threshold, and did not compare raw scores across editions [39]. The item-level documentation improves reproducibility of the operational definition but does not confer construct validity, diagnostic validity, unidimensionality, or measurement invariance.

### 2.6. Covariates

Covariates were selected a priori as characteristics that plausibly precede and influence both dietary behavior and adolescent mental health, and were measured with sufficiently comparable definitions in both surveys. The primary adjustment set therefore focused on sociodemographic, household, family-structure, school, and geographic characteristics: sex, race/skin color, school administrative sector, school location, geographic region, maternal educational attainment, a survey-specific household asset category, and co-residence with the mother and father.

Physical activity, screen time, sleep, body mass index, substance use, meal routines, and psychosocial variables were not included in the primary adjustment set because their temporal position is ambiguous in cross-sectional data; several also overlapped with the 2015 adverse mental health-related indicator score. Residual confounding by behavioral and contextual factors therefore remains possible. Comparable age information beyond the common 13–17 years eligibility band was not retained in the 2015 harmonized analytical file. A 2019-only sensitivity analysis additionally adjusted for age group (13–15 vs. 16–17 years); residual confounding by age remains possible, particularly in 2015.

A study-specific household asset score was constructed separately to represent relative material living conditions. Asset-based measures are widely used as proxies for household socioeconomic position when direct income or expenditure data are unavailable, and similar approaches have been applied in studies using *PeNSE* data [40,41,42]. In 2015, the score included ownership of a landline telephone, mobile phone, computer, internet access, car, and motorcycle, the number of bathrooms, and the presence of a domestic worker. In 2019, the same types of components were used, except that the landline telephone was unavailable and computer ownership was defined as a desktop or notebook computer. Binary indicators contributed 0 or 1 point, whereas bathrooms contributed 0–4 points.

Scores ranged from 0 to 11 in 2015 and from 0 to 10 in 2019, and were classified into lower, intermediate, and higher asset categories: 0–3, 4–6, and 7–11 points in 2015; and 0–3, 4–6, and 7–10 points in 2019. The score was missing when any component was unavailable. Because components and possible ranges differed, categories were derived separately and were not interpreted as directly comparable absolute socioeconomic levels across editions.

Adjusted analyses used complete cases. Missingness was summarized for the primary outcome and each prespecified covariate, and participants included in and excluded from adjusted models were compared using survey-weighted descriptive statistics and absolute standardized differences (Table S2).

### 2.7. Statistical Analysis

Unweighted absolute frequencies described sample selection and retention. Weighted proportions, prevalence estimates, and regression models incorporated the final sampling weights, strata, and primary sampling units supplied by *IBGE*, with Taylor-series linearization [43]. The full survey design was declared before restriction to each analytical domain. Strata containing a single primary sampling unit, including those arising after domain restriction, were handled using the survey package’s grand-mean adjustment. Confidence intervals (CI) and coefficient tests used the *t* distribution with design degrees of freedom calculated as the number of valid primary sampling units minus the number of valid strata. These procedures followed design-based principles for estimation and inference from complex survey samples [43,44].

#### 2.7.1. Primary Association Analyses

Associations between dietary-pattern quartiles and the adverse mental health indicator were estimated using survey-weighted modified Poisson regression with a log link and design-based sandwich variance estimation. This approach directly estimates prevalence ratios (PRs) for binary outcomes and avoids the overestimation that may arise when odds ratios are interpreted as PRs for non-rare outcomes [45,46]. Crude models included only dietary-pattern exposure; adjusted models included all prespecified covariates. Q4 was the reference category, and PRs were reported with 95% CI.

Global associations were evaluated using design-adjusted Wald F tests jointly testing Q1, Q2, and Q3 relative to Q4 [44]. Ordinal trends were evaluated by entering quartiles as a single variable coded 1–4; the resulting PR represented a one-quartile increase in adherence.

Absolute adjusted contrasts were expressed as adjusted prevalence differences (PDs) in percentage points and estimated by marginal standardization. For each adjusted model, predictions were generated for every participant in the adjusted analytical domain, assigning each participant to an adherence quartile while retaining that participant’s observed covariates. The survey-weighted mean predicted prevalence under Q4 was subtracted from the corresponding mean under each comparison quartile. CI incorporated the complex sampling design; PDs were not obtained by algebraic transformation of PRs [47].

For adherence quartile *q*, the adjusted prevalence difference relative to Q4 was calculated by marginal standardization (Equation (1))

(1) $${\hat{PD}}_{q,Q4}=100\left[\frac{\sum_{i\in D}w_{i}{\hat{p}}_{i}\left(q\right)}{\sum_{i\in D}w_{i}}-\frac{\sum_{i\in D}w_{i}{\hat{p}}_{i}\left(Q4\right)}{\sum_{i\in D}w_{i}}\right],\;q\in \{Q1,Q2,Q3\}.$$

Here, D denotes the adjusted analytical domain; $w_{i}$ is the final survey weight for participant i; and $\hat{p}i\left(q\right)$ is the model-predicted probability for that participant after assigning exposure quartile *q* while retaining the observed covariates. The same weighted denominator was used for every counterfactual quartile. Positive and negative PDs indicate higher and lower adjusted prevalence, respectively, relative to Q4.

#### 2.7.2. Continuous-Exposure Analyses

For the continuous-exposure analyses, each survey-specific component score was standardized (Equation (2))

(2) $$Z_{i}=\frac{S_{i}-\bar{S}}{{SD}_{S}}$$

where $S_{i}$ is the component score for participant i, and $\bar{S}$ and ${SD}_{S}$ are the corresponding survey-specific mean and standard deviation. The resulting PR therefore represents the association per one-*SD* increase in the dietary-pattern score.

#### 2.7.3. Between-Survey Comparisons

Between-survey heterogeneity was quantified using the ratio of prevalence ratios (RPR), defined with the 2019 estimate in the numerator and the 2015 estimate in the denominator (Equation (3)). Because the models were fitted separately and the dietary and mental health instruments differed between editions, the RPR compares analogous survey-specific associations and is not an estimate of causal temporal change in an invariant exposure–outcome relationship.

(3) $$\mathrm{RPR}=\frac{{PR}_{2019}}{{PR}_{2015}}$$

In the last equation, ${PR}_{2019}$ and ${PR}_{2015}$ denote the survey-specific prevalence-ratio estimates for the same exposure contrast and model specification. Calculations were performed on the logarithmic scale. Let Δ denote the difference between the 2019 and 2015 log prevalence ratios, and let $v_{y}$ denote the design-based variance of $log({RP}_{y})$ for survey year *y*. Because the surveys were independent, the variance of Δ was the sum of the two survey-specific variances [48], as follows (Equations (4) and (5)):

(4) $$\Delta =\log\left(RPR\right)=\log\left({PR}_{2019}\right)-\log\left({PR}_{2015}\right),$$

(5) $$SE\left(\Delta \right)=\sqrt{v_{2019}+v_{2015}}\;,\;v_{y}=SE{\left\{log\left({PR}_{y}\right)\right\}}^{2}.$$

The 95% CI for the RPR was obtained by exponentiating the Welch–Satterthwaite *t* interval calculated on the logarithmic scale (Equation (6)) [49,50]

(6) $${CI}_{95\%}\left(RPR\right)=exp\left[\Delta \pm t_{0.975,{df}_{WS}}\;SE\left(\Delta \right)\right].$$

An RPR of 1 indicated no difference between survey-specific associations. Individual heterogeneity tests used the Welch–Satterthwaite statistic $t_{WS}$ and degrees of freedom ${df}_{WS}$ (Equation (7)) [49,50], where ${df}_{2019}$ and ${df}_{2015}$ were the survey-specific design degrees of freedom. For categorical quartile models, global between-survey heterogeneity was assessed using a three-degree-of-freedom Wald chi-square test based on the complete covariance matrices for the Q1, Q2, and Q3 contrasts [44]. Comparisons were performed for continuous estimates, ordinal trends, and all three quartile contrasts; no exposure-by-year interaction was fitted to a pooled dataset.

(7) $$t_{WS}=\frac{∆}{\mathrm{SE}(∆)}\;,\;{df}_{WS}=\frac{{\left(v_{2019}+v_{2015}\right)}^{2}}{\frac{v_{2019}^{2}}{{df}_{2019}}+\frac{v_{2015}^{2}}{{df}_{2015}}}.$$

#### 2.7.4. Sensitivity and Robustness Analyses

Primary models were repeated using the alternative mental health indicator, which contrasted the two upper score categories with the two lower categories. Robustness relative to the primary model was summarized descriptively as ΔPR, calculated directly as the sensitivity estimate minus the corresponding primary estimate; no separate hypothesis test was applied.

To assess dependence on dichotomization of the survey-specific adverse mental health-related indicator score, an additional sensitivity analysis treated the total adverse mental health-related indicator score as a continuous outcome. Within each edition, the total score was standardized using the sampling-weighted mean and population-weighted *SD*. Survey-weighted linear regression models with design-based variance estimation were fitted using the same adjusted analytical domain and the same prespecified covariates as the primary models [43]. Dietary-pattern adherence was evaluated both by quartiles, with Q4 as the reference, and continuously per one-*SD* increase in the corresponding PCA score. Regression coefficients represent adjusted mean differences in the survey-specific adverse mental health-related indicator score expressed in outcome *SD* units. Because the score composition and measurement ranges differed between editions, coefficients were interpreted only within each survey and were not used to infer temporal change or measurement equivalence.

To assess sensitivity to unequal selection probabilities during dietary-pattern derivation, the manuscript association models were re-estimated using scores and weighted empirical quartiles from the final-weighted PCA solutions. Agreement between weighted and unweighted solutions was evaluated using Tucker congruence of the aligned loading vectors [51], score correlations, explained variance, and nutritional interpretation.

Sensitivity to potential unmeasured confounding was assessed using E-values for the adjusted Q1 vs. Q4 PRs and the 95% CI limit closest to 1. For either a point estimate or a confidence limit, the ratio was first transformed to ${PR}_{E}\geq 1$ (Equation (8)). As a sensitivity analysis for complete-case selection, stabilized inverse-probability-of-complete-case weights were estimated separately by survey and multiplied by the original sampling weights; the focal Q1 vs. Q4 models were then re-estimated using the original strata and primary sampling units [52]. This analysis cannot identify or eliminate nonignorable missingness.

(8) $${PR}_{E}=\max\left(PR,\frac{1}{PR}\right).$$

The corresponding E-value was then calculated as (Equation (9))

(9) $$E\text{-}\mathrm{value}={PR}_{E}+\sqrt{{PR}_{E}\left({PR}_{E}-1\right)}.$$

In the last equation, PR denotes either the adjusted point estimate or the confidence limit being evaluated, and ${PR}_{E}$ denotes its transformed value on a scale of at least 1. The same calculation was applied to the 95% CI limit closest to 1 when the interval excluded the null; when the interval included 1, the confidence-limit E-value was set to 1.00. The E-value represents the minimum strength of association, on the prevalence-ratio scale, that an unmeasured confounder would need to have with both exposure and outcome, beyond the measured covariates, to explain the observed association [53].

All tests were two-sided, with *α* = 0.05. Exact *p*-values were reported to three decimal places, and values below 0.001 were reported as *p* < 0.001. The four adjusted global quartile tests (two dietary patterns in each survey) formed the primary inferential family and were evaluated with Holm’s procedure [54]. Q1 vs. Q4 contrasts were retained as focal effect-size comparisons; other quartile contrasts, trends, continuous models, between-survey comparisons, and sensitivity analyses were interpreted as supportive or exploratory.

### 2.8. Statistical Software

All analyses, including primary data management, PCA derivation and diagnostics, complex-survey regression, and robustness and sensitivity analyses—Horn’s parallel analysis, continuous-outcome models, 2019 age-group adjustment, and complete-case weighting—were conducted in R version 4.6.0 (R Foundation for Statistical Computing, Vienna, Austria; [55]).

The complex sampling design was specified and analyzed using the survey package, version 4.5 [43,56]. Survey-weighted correlation and covariance matrices, weighted prevalence estimates, complex-survey regression models, design-adjusted Wald tests, and model-based predictions were obtained within this framework. Supplementary weighted component analyses, sampling-weighted Cronbach’s *α* estimates, marginal standardization, between-survey RPR estimates, ΔPR, and E-values were calculated from unrounded estimates using prespecified R functions. E-values were calculated from the adjusted PR estimates and their confidence limits [53].

### 2.9. Ethical Considerations

The 2015 and 2019 editions of the *PeNSE* were reviewed and approved by the Brazilian National Research Ethics Commission (*Comissão Nacional de Ética em Pesquisa*—*CONEP*), the highest-level body within Brazil’s research ethics review system (Protocol *CAAE* No. 38990714.6.0000.0008). *PeNSE* 2015 was approved through Opinion No. 1006467 (30 March 2015), and *PeNSE* 2019 was approved through Opinion No. 3249268 (8 April 2019). Participation was voluntary, and confidentiality was ensured [22,23]. This study used only anonymized, publicly available secondary data; therefore, no additional informed consent was required.

### 2.10. Reporting Guideline

This report was prepared in accordance with the Strengthening the Reporting of Observational Studies in Epidemiology recommendations for cross-sectional studies [57] (STROBE Checklist S1).

## 3. Results

### 3.1. Sample Selection and Analytical Samples

After age and dietary-data restrictions, the PCA samples comprised 10,835 participants in 2015 and 124,194 in 2019; adjusted models included 8299 (76.6%) and 101,353 (81.6%) participants, respectively (Table 1). Missing maternal educational attainment was the main source of exclusion (21.6% in 2015 and 15.8% in 2019, unweighted). Included vs. excluded comparisons showed modest imbalances in several observed characteristics; detailed weighted missingness, standardized differences, and complete-case sensitivity results are provided in Table S2.

### 3.2. Participant Characteristics

Weighted participant characteristics are summarized in Table 2. In both editions, the sex and skin-color distributions were similar, and most participants attended public, urban schools. The main between-edition differences concerned maternal educational attainment and household asset categories.

### 3.3. Internal Consistency of the Survey-Specific Adverse Mental Health-Related Indicator Scores

Internal consistency of the survey-specific adverse mental health-related indicator scores was modest in 2015 (Cronbach’s *α* = 0.564; weighted *α* = 0.548) and higher in 2019 (*α* = 0.738; weighted *α* = 0.737). The weighted prevalence of membership in the primary adverse-score category was 19.5% in 2015 and 24.3% in 2019. The complete item composition and adverse-oriented recoding are presented in Table S1.

### 3.4. Dietary Patterns Identified by PCA

**Figure 1:**
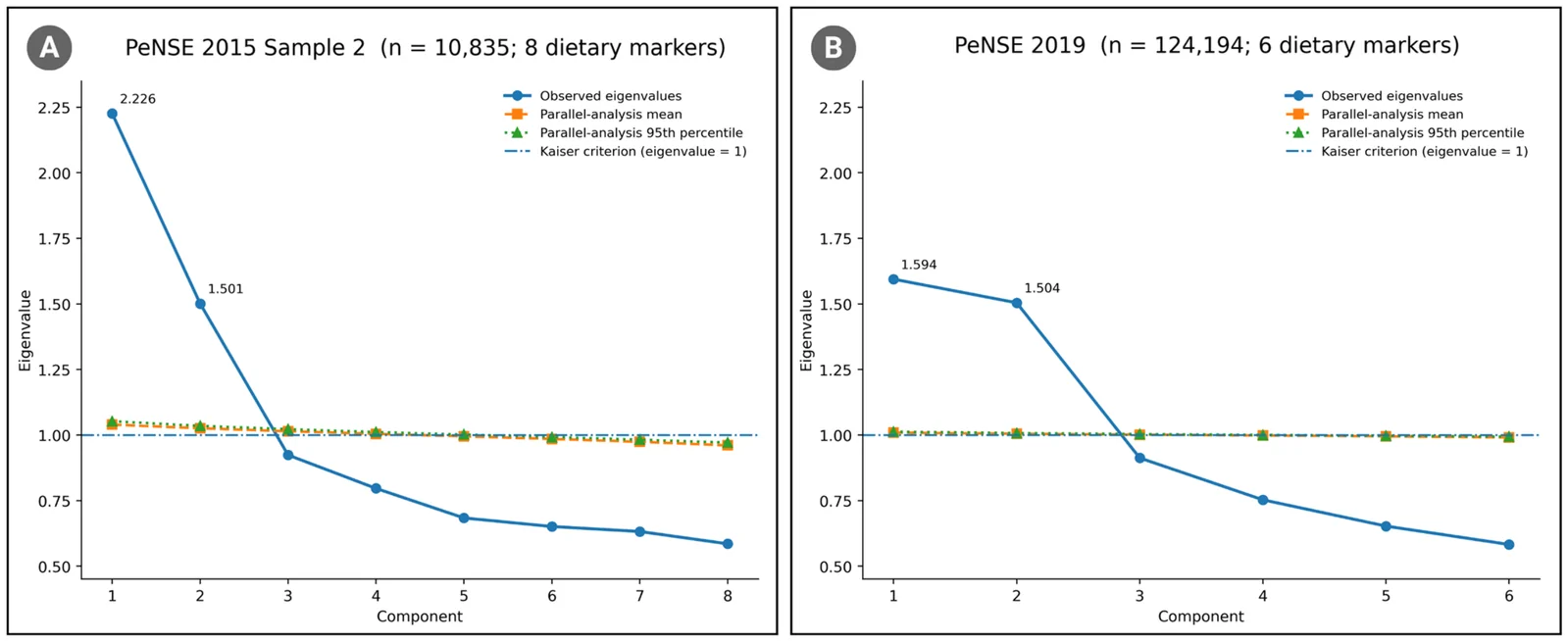
Scree plots and Horn parallel-analysis results for dietary-pattern component retention in *PeNSE* 2015 and 2019. (**A**) *PeNSE* 2015 Sample 2, based on eight dietary markers; (**B**) *PeNSE* 2019, based on six dietary markers. Note: Observed eigenvalues were obtained from the primary unweighted Pearson correlation matrices. Horn parallel analysis used 5000 random correlation matrices with the same sample size and number of markers as each survey, with the 95th percentile used as the retention threshold. Two components were retained in both editions because only the first two observed eigenvalues exceeded the corresponding random thresholds. The horizontal dotted line indicates the Kaiser eigenvalue threshold of 1.

Two components were retained in each edition. The first three eigenvalues were 2.226, 1.501, and 0.924 in 2015 and 1.594, 1.504, and 0.913 in 2019; scree plots and Horn parallel analysis supported retention of two components in both surveys (Figure 1A for *PeNSE* 2015 and Figure 1B for *PeNSE* 2019). KMO was 0.716 in 2015 and 0.581 in 2019, and the retained components explained 46.6% and 51.6% of total variance, respectively. Weighted and unweighted solutions were nearly identical (score correlations 0.9993–0.9998 and Tucker congruence 0.9987–0.9999) (Table 3).

### 3.5. Dietary-Pattern Adherence Quartiles and the Adverse Mental Health Indicator

Table 4 presents crude and adjusted relative and absolute associations between dietary-pattern quartiles and the adverse mental health indicator, with Q4 as the reference.

**Figure 2:**
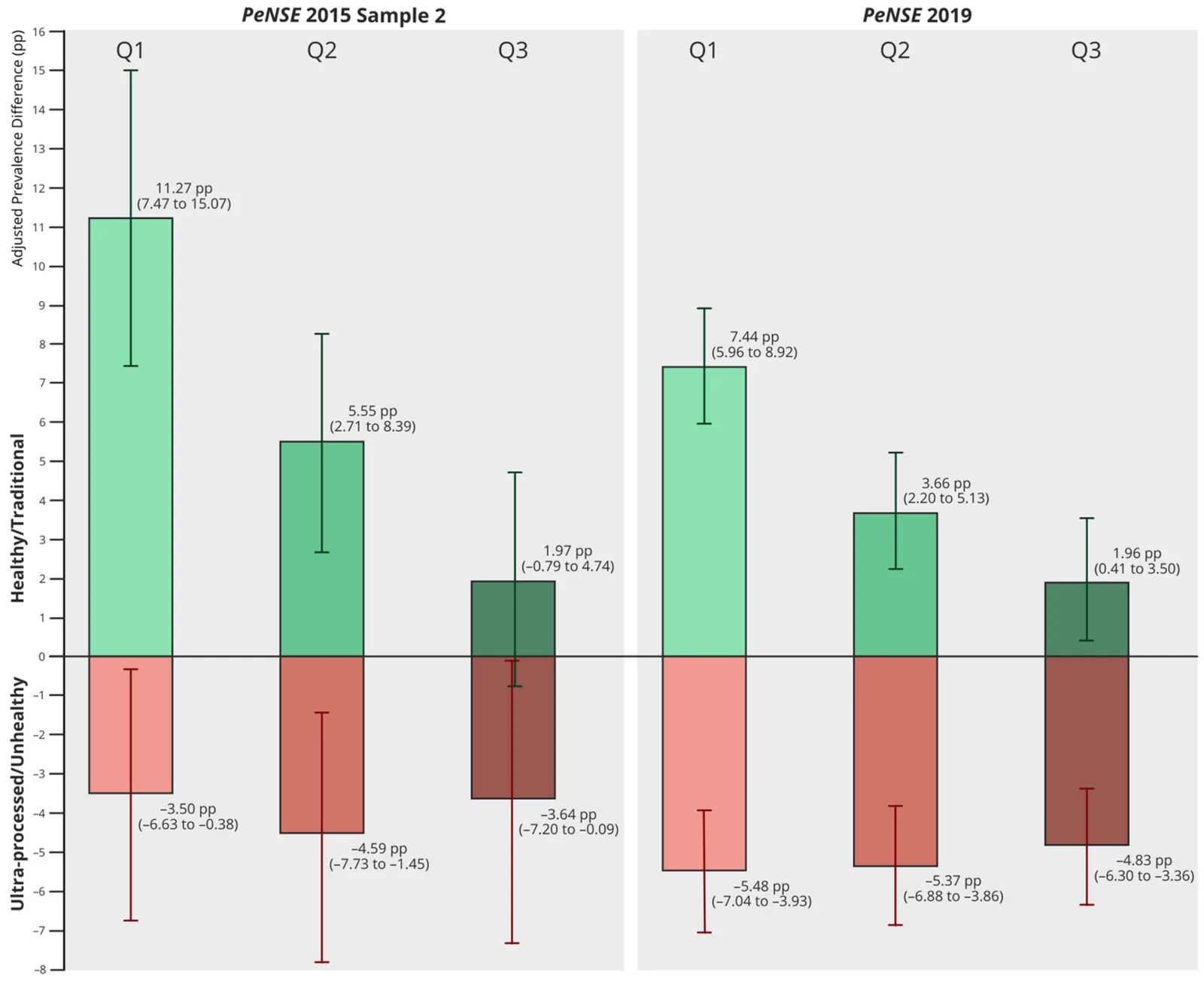
Adjusted prevalence differences in the survey-specific adverse mental health indicator across dietary-pattern adherence quartiles among adolescents aged 13–17 years.

Compared with Q4, Q1 of the ultra-processed/unhealthy pattern was associated with a lower adjusted prevalence in 2015 (PR = 0.85; PD = −3.50 percentage points) and 2019 (PR = 0.81; PD = −5.48 percentage points). Conversely, Q1 of the healthy/traditional pattern was associated with a higher adjusted prevalence in 2015 (PR = 1.75; PD = 11.27 percentage points) and 2019 (PR = 1.34; PD = 7.44 percentage points). The adjusted prevalence differences and their 95% CI are displayed in Figure 2. Global associations and ordinal trends were significant for both patterns in both editions, supporting graded relationships. For the adjusted 2015 Q3 vs. Q4 contrast in the ultra-processed/unhealthy pattern, the unrounded upper confidence limit was below 1.000; it is displayed as <1.000 to avoid an apparent inconsistency with *p* = 0.045. Holm correction of the four primary global quartile tests did not change the inferential conclusions; the 2015 ultra-processed/unhealthy test remained *p* = 0.025, and the other three remained below 0.004 under a conservative bound.

Prevalence differences were estimated by marginal standardization and expressed as percentage points relative to Q4, the highest relative adherence group within each survey edition. Negative values indicate lower and positive values higher adjusted prevalence than Q4. Because quartiles, dietary patterns, and outcome indicators were survey-specific, differences between editions represent between-survey heterogeneity rather than absolute temporal change. Points show adjusted prevalence differences and whiskers show 95% CI; colors and marker shapes provide redundant visual coding.

### 3.6. Secondary and Between-Survey Analyses

Table 5 summarizes the adjusted continuous, ordinal, categorical, and between-survey comparisons.

Continuous and ordinal estimates supported the quartile-based findings. No between-survey heterogeneity was detected for the ultra-processed/unhealthy pattern (global quartile *p* = 0.903). The healthy/traditional association estimates differed between surveys and were larger in magnitude in 2015 for the continuous score (RPR = 1.13; *p* = 0.001), ordinal trend (RPR = 1.10; *p* = 0.004), and Q1 vs. Q4 contrast (RPR = 0.77; *p* = 0.008), with global quartile heterogeneity (*p* = 0.033). Because the constructs were survey-specific, these findings indicate between-survey heterogeneity in survey-specific associations and do not represent causal or absolute temporal change in identical measures.

### 3.7. Results of Sensitivity and Robustness Analyses

Table 6 and Table 7 summarize the alternative-outcome, E-value, and weighted-PCA analyses.

The alternative outcome and weighted-PCA analyses preserved the direction and principal statistical significance of the associations, and weighted-PCA estimates differed minimally from the primary estimates. Point-estimate E-values ranged from 1.65 to 2.89, indicating varying sensitivity to potential unmeasured confounding. Overall, the main conclusions were not materially dependent on the outcome threshold or PCA weighting.

Additional sensitivity analyses also supported the robustness of the findings. Complete-case weighting produced focal Q1 vs. Q4 PRs within 0.015 of the primary estimates in both surveys, with no extreme stabilized selection factors (Table S2). Treating the survey-specific adverse mental health-related score as a continuous standardized outcome preserved the direction of association for both dietary patterns in both surveys, indicating that the findings were not dependent on outcome dichotomization (Table S1).

Further adjustment for age group in 2019 changed all six quartile PRs by less than 0.01, indicating no material impact on the estimates. Continuous-outcome, age-adjusted, alternative-outcome, weighted-PCA, E-value, complete-case-weighted, and between-survey analyses were interpreted as supportive or exploratory, and no substantive conclusion depended on an isolated secondary *p*-value.

## 4. Discussion

### 4.1. Main Findings and Measurement Interpretation

Across two nationwide school-based surveys, we identified ultra-processed/unhealthy and healthy/traditional dietary patterns that were consistently associated with the survey-specific adverse mental health indicator. Higher adherence to the ultra-processed/unhealthy pattern and lower adherence to the healthy/traditional pattern were associated with a higher prevalence of membership in the adverse-score category in both editions, and the agreement among categorical, continuous, and ordinal estimates supports graded rather than isolated category-specific associations. The ultra-processed/unhealthy associations were similar across surveys, whereas the healthy/traditional association was stronger in 2015. These results should be interpreted as associations within two independent survey-specific measurement systems, not as evidence of temporal change in an identical dietary-pattern–mental-health-score relationship.

The ultra-processed/unhealthy findings align with evidence from *PeNSE* 2019 showing that consumption of a greater number of ultra-processed food types during the previous 24 h was associated with more frequent mental health symptoms in both sexes [20]. The present analysis extends that evidence by using seven-day, PCA-derived adherence scores and survey-specific score-based adverse mental health-related outcomes rather than a count-based 24 h exposure and five-symptom score. The corresponding Q1 vs. Q4 adjusted prevalence differences were −3.50 percentage points in 2015 and −5.48 percentage points in 2019. Broader meta-analytic evidence also indicates an association between greater ultra-processed food consumption and depressive and anxiety symptoms, although the available synthesis included predominantly adult populations and cross-sectional studies [15]. Reviews focused specifically on adolescents report a generally similar direction of association while emphasizing substantial heterogeneity and the limited strength of causal evidence [16,17]. Thus, the present findings add nationally representative, pattern-based evidence without establishing causality.

The healthy/traditional pattern showed a complementary association, with adjusted prevalence differences of 11.27 percentage points in 2015 and 7.44 percentage points in 2019 between Q1 and Q4. This direction is consistent with prospective adolescent evidence suggesting that healthier dietary patterns are often associated with fewer depressive or broader mental health symptoms, although findings remain heterogeneous [16]. Interpretation should remain specific to the component identified here, which primarily reflected beans, vegetables or leafy greens, and fresh fruit rather than a comprehensive index of total diet quality. Brazilian adolescents occupy heterogeneous dietary and nutritional profiles embedded in socioeconomic, family, school, behavioral, and psychosocial contexts [24], and a regional study found lower interleukin-6 concentrations with greater adherence to a prudent pattern [13]. These results support plausibility but do not make empirically derived pattern labels interchangeable across studies.

The outcome should be interpreted as a relative, non-diagnostic, score-based indicator of adverse mental health-related experiences and symptoms within each survey, rather than as a measure of mental health itself or of a specific disorder. Its items capture a broad mixture of internalizing symptoms, perceived social support or connectedness, peer adversity, and, in 2015, sleep disruption related to worry. The 2019 coefficient indicates moderate internal consistency, whereas the lower 2015 coefficient suggests a more heterogeneous multidimensional summary. This does not, by itself, preclude epidemiological ranking in the 2015 survey, but it limits construct-specific interpretation and may have introduced measurement error. Cronbach’s *α* does not establish unidimensionality, construct validity, criterion validity, or diagnostic accuracy. Likewise, consistency under the broader outcome threshold supports robustness to categorization rather than psychometric validation. Because item content, recall periods, and score ranges differed, between-survey comparisons concern the magnitude of survey-specific associations and no change in an invariant measure of mental health [39].

The continuous-outcome sensitivity analysis reduced dependence on the primary distribution-based cutoff and preserved the same substantive direction for both dietary patterns in each edition. This convergence supports robustness to outcome dichotomization, but it does not establish unidimensionality, construct validity, criterion validity, diagnostic accuracy, or measurement invariance of the survey-specific adverse mental health-related indicator scores.

The 2019 PCA warrants cautious interpretation because the KMO value (0.581 unweighted; 0.585 weighted) indicates marginal common variance among the available markers. Nevertheless, the scree plot, Horn parallel analysis, explained variance, coherent loadings, and near-identical weighted and unweighted solutions supported a parsimonious two-component representation. These diagnostics support retention but do not establish a strongly coherent or metrically invariant latent dietary construct.

### 4.2. Bidirectionality, Causal Interpretation, and Residual Confounding

Several biological, behavioral, and contextual pathways may jointly contribute to the observed associations. Diet quality may relate to nutrient adequacy, systemic inflammation, oxidative stress, neuroplasticity, and microbiota–gut–brain signaling [11,12]. The inverse association between a prudent pattern and interleukin-6 among older Brazilian adolescents is compatible with an inflammatory pathway, although neither that study nor the present analysis demonstrates mediation [13]. Shared determinants are also important: screen time, private-school attendance, female sex, and higher body mass index recur as correlates of ultra-processed food consumption [8], while multiple risk behaviors and their socioeconomic inequalities cluster across family and school contexts [25]. Emotional distress may also alter appetite, meal regularity, and preferences for highly palatable foods. The findings are therefore compatible with bidirectionality, residual confounding, and behavioral clustering rather than a single causal pathway.

Plausible pathways operate in both directions. Greater adherence to the healthy/traditional pattern and lower adherence to the ultra-processed/unhealthy pattern may relate to the mental health-related experiences and symptoms represented in the survey-specific adverse mental health-related indicator scores through nutrient adequacy, inflammatory and oxidative pathways, neuroplasticity, hypothalamic–pituitary–adrenal axis regulation, tryptophan metabolism, and microbiota–gut–brain signaling [11,12,13]. Conversely, sadness, worry, irritability, perceived lack of care, peer adversity, and sleep disruption may reduce planning and meal regularity, alter appetite and reward sensitivity, and increase reliance on convenient, highly palatable foods or emotional eating. Experimental and ecological studies of adolescents show that negative affect, childhood adversity, and responsiveness to external food cues can shape eating behavior, supporting the plausibility of the reverse pathway [58,59].

Shared determinants—including socioeconomic adversity, food insecurity, family stress, peer experiences, screen exposure, and school food environments—may also generate both dietary profiles and higher survey-specific adverse mental health-related indicator scores. In Brazilian adolescents, adverse childhood experiences such as domestic violence, parental separation, and physical or emotional neglect have also been associated with nutrition-related outcomes, reinforcing the potential relevance of adversity as a common contextual determinant [60]. The observed cross-sectional prevalence ratios may therefore reflect a mixture of diet-to-experience or symptom pathways, reverse pathways from emotional or psychosocial experiences to dietary behavior, and common causes; the study cannot separate their relative contributions.

Between-survey heterogeneity was not detected for the ultra-processed/unhealthy pattern, whereas the healthy/traditional association estimates were larger in magnitude in 2015. This difference may reflect the surveys’ distinct dietary markers, mental health items, score ranges, and sampling domains rather than a temporal weakening of a common, invariant effect. Accordingly, the RPRs quantify heterogeneity between survey-specific associations and should not be interpreted as changes in metrically equivalent exposure–outcome relationships. *PeNSE*-based evidence also indicates that dietary and clustered risk-behavior profiles are socially patterned and may vary across contexts and survey periods [24,25].

The robustness analyses reinforced the main interpretation. Broadening the score-based definition of the adverse indicator and deriving patterns using survey-weighted correlation matrices preserved the direction and principal significance of the associations. The E-values indicated that unmeasured confounding of modest to moderate magnitude could attenuate some estimates, particularly the 2015 ultra-processed/unhealthy contrast, whereas stronger joint associations would be required to explain the healthy/traditional estimates. These analyses improve transparency but do not eliminate residual confounding.

Adjustment for multiplicity across the four primary global tests preserved the overall inferential pattern. Nevertheless, the manuscript contains several secondary contrasts and robustness analyses; isolated nominal *p*-values, particularly those close to 0.05, should not be interpreted as independent confirmatory findings. Greater weight should be placed on effect magnitude, precision, the global tests, and convergence across prespecified analytical specifications.

The close agreement between the primary and IPCCW focal estimates suggests that the main associations were not materially altered after reweighting complete cases for selection patterns captured by the observed variables in the propensity model. This finding reduces, but does not remove, concern about complete-case selection. The analysis cannot establish the missing-data mechanism, recover information under missing-not-at-random processes, or account for determinants of selection that were unmeasured or incorrectly modeled.

The adjustment strategy was designed to estimate associations after accounting for comparable sociodemographic, household, family-structure, school, and geographic characteristics, rather than conditioning on every available variable. This distinction matters because physical activity, screen time, sleep, body mass index, substance use, meal routines, and psychosocial experiences may occupy different positions in the causal system and could be mediators, consequences, or colliders in addition to potential shared causes. Nevertheless, residual confounding remains plausible from age within the restricted 13–17-year range, food insecurity, violence and adversity, behavioral clustering, and detailed school or neighborhood food environments. The E-values quantify the strength that a single unmeasured confounder would need to have with exposure and outcome to explain selected estimates, but they do not exclude several weaker confounders acting jointly and do not address measurement error or complete-case selection.

The 2019 age-group sensitivity analysis further showed that adjustment for the available 13–15 vs. 16–17-year grouping did not materially alter the dietary-pattern estimates. This supports robustness to broad age-group adjustment within the 2019 survey. However, it does not eliminate residual age confounding in 2015 and should not be interpreted as evidence that single-year age is irrelevant.

### 4.3. Implications for School Food and Mental Health Policies

These findings support integrated nutrition surveillance and population-level monitoring of mental health-related experiences and symptoms in schools. However, they also indicate that policy responses should extend beyond individual food choice. Evidence on Brazilian adolescents points to family and school relationships, socioeconomic inequality, screen-based sedentary behavior, and school context as correlates of mental health, ultra-processed food consumption, or clustered risk behaviors [8,25]. Strategies that improve the availability and acceptability of fresh or minimally processed foods, reduce routine exposure to ultra-processed products, and strengthen supportive family and school environments may therefore align with broader adolescent health promotion. This supports multisectoral adolescent health promotion integrating healthy eating, health education, equity, and school–health service coordination [61]. Nevertheless, adolescent intervention evidence remains limited and heterogeneous [16], and the present cross-sectional analyses cannot establish that changing dietary patterns would reduce membership in the adverse-score category, improve adolescents’ mental health, or identify the responsible food components.

Policy implications should emphasize integrated prevention and health promotion rather than treating dietary change as a proven mental health therapy. The findings are consistent with the strengthening implementation of Brazil’s National School Feeding Program (*Programa Nacional de Alimentação Escolar*—*PNAE*). Under the current regulatory framework consolidated by Resolution *CD*/*FNDE* No. 4/2026, at least 85% of federal *PNAE* resources must be allocated to *in natura* or minimally processed foods, while no more than 10% may be allocated to processed and ultra-processed products [62]. Operational priorities include culturally appropriate menus that regularly offer beans, fruits, and vegetables; procurement from family farms; food and nutrition education integrated into the curriculum; and local measures to reduce access to and promotion of ultra-processed products in and around schools. These actions align with Brazilian food policies that promote minimally processed foods, regional traditions, family farming, sustainability, and equitable access [63]. Their implementation should also account for regional feasibility and food insecurity, rather than relying solely on individual choice.

Nutrition actions should be linked to school-based mental health promotion, including population surveillance, mental health literacy, supportive peer relationships, anti-bullying initiatives, and clear referral pathways to primary and psychosocial care. The score-based adverse mental health-related indicator used in this study is not suitable for individual screening or diagnosis; its appropriate policy role is population-level monitoring and prioritization. An umbrella review found that multicomponent school-based dietary interventions—combining education with changes in food availability and family or community involvement—were more promising than isolated information campaigns, although most evidence came from high-income countries and Brazilian implementation, feasibility, and equity effects require evaluation [64].

Accordingly, the present results support coordinated surveillance and prevention strategies addressing both food environments and adolescent well-being. They do not identify a specific causal food component, establish that dietary modification would reduce the adverse score-based indicator or improve mental health, or justify replacing mental health assessment and care with nutrition interventions. Longitudinal studies with repeated, validated mental health measures, more comprehensive dietary assessment, and explicit causal models are needed to clarify temporality, mechanisms, and the effects of modifiable school and household environments.

### 4.4. Limitations and Strengths

Several limitations should be acknowledged. The cross-sectional design precludes establishing temporal sequence and permits reverse causation. Dietary markers, mental health-related items, and covariates were self-reported and may be affected by recall, interpretation, reporting, and social-desirability biases. The survey-specific composite scores of adverse mental health-related indicators were not validated measures of a unitary mental health construct or diagnostic scales, differed in item composition, recall periods, and score ranges, and showed modest internal consistency in 2015 (unweighted and weighted Cronbach’s *α* = 0.564 and 0.548, respectively, versus 0.738 and 0.737 in 2019). These scores were designed as survey-specific epidemiological indicators of relative mental health-related burden, not as psychometric or diagnostic scales; therefore, construct-specific interpretation remains limited without formal validation. The distribution-based thresholds identified the relatively most adverse segment of each survey-specific adverse mental health-related indicator score distribution rather than clinical cutoffs or disorder prevalence, and ties prevented exact 25% groups. The simple sum assigned one point for each response-category step; because the component items had different response ranges, five-category items had greater possible influence on the total score than binary or four-category items. Item-level coding is reported transparently in Table S1, but factorial structure, construct validity, criterion validity, diagnostic accuracy, and measurement invariance were not established.

The continuous-score sensitivity analysis treated each bounded, discrete adverse mental health-related indicator score as approximately continuous. Although design-based variance estimation and large sample sizes support its use as a robustness analysis of average score differences, this modeling choice does not convert these scores into validated interval-level scales and does not make their coefficients directly comparable across survey editions.

Dietary patterns were derived from a limited, edition-specific set of frequency markers rather than a comprehensive dietary assessment. Because PCA is data-driven, the component solutions and labels depend on marker availability, preprocessing, retention criteria, and substantive interpretation [10]. Although retention diagnostics supported two components in each survey, the patterns, quartiles, and mental health indicators were not metrically equivalent across editions. Accordingly, between-survey estimates reflect heterogeneity in analogous relative associations rather than temporal change in invariant constructs, consistent with the measurement heterogeneity emphasized in adolescent reviews [16,17]. Complete-case restriction excluded 23.4% of PCA-eligible participants in 2015 and 18.4% in 2019, mainly because maternal educational attainment was missing, and observed differences between included and excluded participants indicate that selection bias remains possible despite the weighting sensitivity analysis. Residual confounding by age, physical activity, screen exposure, food insecurity, adversity, and other unmeasured contextual factors also cannot be excluded. Secondary analyses were interpreted as supportive or exploratory because of multiplicity.

Important strengths include the use of large, nationwide, school-based probability samples and restriction of the 2015 analysis to Sample 2 to improve age-range comparability with *PeNSE* 2019. All inferential analyses incorporated survey weights, strata, and primary sampling units. Dietary patterns were derived separately for each edition and evaluated through categorical, continuous, and ordinal specifications, while adjusted prevalence differences complemented relative measures. Formal heterogeneity tests and robustness analyses using an alternative outcome, weighted PCA, and E-values addressed between-edition variation, threshold choice, PCA weighting, and potential unmeasured confounding. The convergence of relative, absolute, continuous, ordinal, and sensitivity estimates reduces the likelihood that the principal findings were artifacts of a single categorization or modeling decision.

## 5. Conclusions

Among school-attending Brazilian adolescents aged 13–17 years, the 2015 and 2019 editions of the *PeNSE* each yielded two dietary patterns: an ultra-processed/unhealthy pattern and a healthy/traditional pattern. In both surveys, greater adherence to the ultra-processed/unhealthy pattern and lower adherence to the healthy/traditional pattern were associated with a higher prevalence of the survey-specific adverse mental health indicator. No between-survey heterogeneity was detected for the ultra-processed/unhealthy pattern, whereas the healthy/traditional estimates differed in magnitude between surveys. These comparisons reflect heterogeneity between survey-specific associations and cannot be interpreted as causal or absolute temporal change because the dietary and mental health measures were not invariant across editions. These findings identify and quantify the associations specified in the study objective and support integrated nutrition surveillance and population-level monitoring of mental health-related experiences and symptoms, but they do not establish temporality or causality, nor do they indicate that modifying dietary patterns would reduce the adverse score-based indicator or improve mental health.

## Figures and Tables

**Table 1:** Sample selection and analytical domains for school-attending Brazilian adolescents aged 13–17 years, by sex.

Selection Stage	*PeNSE* 2015 Sample 2			*PeNSE* 2019			
	Total (*n*)	Male	Female	Total (*n*)	Male	Female	NR
Initial retained records	16,556	8287	8269	165,838	78,011	80,788	7039
Questionnaire data available	16,556	8287	8269	159,245	78,011	80,788	446
Eligible, aged 13–17 years	10,926	5522	5404	124,898	61,462	63,148	288
Included in unweighted PCA estimation	10,835	5468	5367	124,194	60,994	62,912	288
Included in weighted PCA estimation	10,835	5468	5367	124,194	60,994	62,912	288
Unweighted-PCA crude model domain	10,544	5283	5261	123,018	60,155	62,575	288
Unweighted-PCA adjusted model domain	8299	4086	4213	101,353	48,537	52,816	0
Weighted-PCA crude model domain	10,544	5283	5261	123,018	60,155	62,575	288
Weighted-PCA adjusted model domain	8299	4086	4213	101,353	48,537	52,816	0

Note: *n* represents unweighted absolute frequencies; NR, sex not reported; PCA, principal component analysis. PCA-estimation rows require age eligibility and complete survey-specific dietary markers; the weighted PCA additionally requires a valid final weight. Model domains also require a valid primary outcome and complete survey design; adjusted domains additionally require all nine prespecified covariates. In 2019, NR participants were retained in the PCA estimation and crude models but were excluded from the adjusted models because sex was an adjustment covariate.

**Table 2:** Weighted characteristics of school-attending Brazilian adolescents aged 13–17 years.

Characteristic/Category	*PeNSE* 2015 Sample 2		*PeNSE* 2019	
	*n*	Weighted % (95% CI)	*n*	Weighted % (95% CI)
Sex	10,926		124,898	
Male	5522	50.3 (48.6–51.9)	61,462	49.3 (48.5–50.1)
Female	5404	49.7 (48.1–51.4)	63,148	50.7 (49.9–51.5)
Not reported	—	—	288	0.1 (0.0–0.1)
Skin color/ethnicity	10,918		122,366	
White	4300	36.2 (34.4–38.0)	47,498	36.0 (35.1–36.9)
Black	1277	13.2 (12.0–14.6)	13,542	13.6 (13.1–14.1)
Asian/Yellow	463	4.1 (3.6–4.7)	4401	3.7 (3.5–4.0)
Brown	4560	43.6 (41.6–45.6)	53,413	43.5 (42.7–44.4)
Indigenous	318	2.9 (2.4–3.5)	3512	3.1 (2.9–3.4)
Geographic region	10,926		124,898	
North	2139	9.2 (7.7–10.9)	28,264	10.8 (10.0–11.8)
Northeast	2277	28.6 (25.9–31.4)	42,955	28.4 (27.1–29.6)
Southeast	2083	41.0 (37.9–44.2)	22,600	38.8 (37.2–40.4)
South	2152	13.6 (12.1–15.2)	13,439	13.7 (12.9–14.6)
Central-West	2275	7.6 (6.8–8.6)	17,640	8.3 (7.8–8.8)
School administrative sector	10,926		124,898	
Public	8287	87.1 (84.8–89.0)	65,073	85.5 (84.7–86.3)
Private	2639	12.9 (11.0–15.2)	59,825	14.5 (13.7–15.3)
School location	10,926		124,898	
Urban	10,439	94.5 (91.8–96.3)	118,642	92.4 (91.0–93.6)
Rural	487	5.5 (3.7–8.2)	6256	7.6 (6.4–9.0)
Maternal educational attainment	8552		105,046	
0–8 years	2348	32.7 (30.3–35.2)	23,954	36.9 (35.8–38.1)
9–11 years	3437	43.1 (41.0–45.3)	32,098	34.1 (33.3–35.0)
≥12 years	2767	24.2 (21.6–27.0)	48,994	28.9 (28.1–29.8)
Household asset category	10,881		124,621	
Lower	1721	18.6 (16.7–20.5)	19,725	23.2 (22.2–24.2)
Intermediate	5441	53.0 (51.0–55.0)	68,185	60.6 (59.8–61.4)
Higher	3719	28.4 (26.1–30.9)	36,711	16.2 (15.6–16.9)
Lives with mother	10,919		124,835	
No	1259	11.6 (10.7–12.5)	13,885	11.9 (11.5–12.3)
Yes	9660	88.4 (87.5–89.3)	110,950	88.1 (87.7–88.5)
Lives with father	10,918		124,791	
No	4100	38.4 (36.9–39.9)	46,190	39.6 (38.8–40.4)
Yes	6818	61.6 (60.1–63.1)	78,601	60.4 (59.6–61.2)

Note: 95% CI, 95% confidence interval; *n* represents unweighted category frequencies; weighted percentages and 95% CI account for sampling weights, strata, and primary sampling units. Estimates were calculated among age-eligible participants with a valid survey design and a valid value for the corresponding characteristic; denominators may therefore vary across characteristics. Sex not reported is presented descriptively but was not included in the adjusted models.

**Table 3:** Rotated component loadings and communalities from the primary unweighted and survey-weighted PCA among school-attending Brazilian adolescents aged 13–17 years.

Dietary Marker	*PeNSE* 2015 Sample 2			*PeNSE* 2019		
	Ultra- Processed/ Unhealthy	Healthy/ Traditional	*h* ^2^	Ultra- Processed/ Unhealthy	Healthy/ Traditional	*h* ^2^
Primary unweighted PCA						
Beans	−0.028	0.522	0.273	−0.100	0.481	0.241
Vegetables or leafy greens	−0.037	0.796	0.635	−0.001	0.808	0.654
Fresh fruit	0.099	0.767	0.598	0.088	0.786	0.625
Soft drinks	0.670	−0.079	0.456	0.742	−0.113	0.563
Sweets	0.638	0.039	0.408	0.700	0.017	0.491
Ultra-processed foods	0.687	0.011	0.472	—	—	—
Savory snacks	0.706	0.006	0.498	—	—	—
Snacks	0.621	0.042	0.387	—	—	—
Fast food	—	—	—	0.724	0.029	0.525
Survey-weighted PCA						
Beans	−0.019	0.506	0.256	−0.127	0.481	0.248
Vegetables or leafy greens	−0.026	0.802	0.643	0.033	0.805	0.649
Fresh fruit	0.115	0.767	0.601	0.131	0.778	0.622
Soft drinks	0.678	−0.082	0.466	0.761	−0.087	0.587
Sweets	0.633	0.052	0.403	0.702	0.013	0.493
Ultra-processed foods	0.690	0.026	0.477	—	—	—
Savory snacks	0.705	0.022	0.498	—	—	—
Snacks	0.619	0.056	0.387	—	—	—
Fast food	—	—	—	0.711	0.052	0.509

Note: *h*^2^, communality, calculated as the sum of squared loadings across the two retained components; PCA, principal component analysis. Both primary and weighted solutions used Pearson correlation matrices, two retained components, Varimax rotation with Kaiser normalization, and regression scoring. Weighted PCA used final sampling weights only. Component order and sign were aligned so that higher scores indicate greater adherence to the named pattern. Weighted–unweighted score correlations ranged from 0.9993 to 0.9998, and Tucker loading-congruence coefficients ranged from 0.9987 to 0.9999. Em dashes indicate markers unavailable in the respective survey.

**Table 4:** Relative and absolute associations between primary unweighted PCA dietary-pattern quartiles and the survey-specific adverse mental health indicator among school-attending Brazilian adolescents aged 13–17 years.

Dietary Pattern and Measure	*PeNSE* 2015 Sample 2						*PeNSE* 2019					
	Q1	Q2	Q3	Q4	Global *p*-Value *	*p* for Trend ^†^	Q1	Q2	Q3	Q4	Global *p*-Value *	*p* for Trend ^†^
Ultra-processed/unhealthy dietary pattern												
Crude PR	**0.83**	**0.84**	0.86	1.00 (ref.)	0.050	**0.014**	**0.70**	**0.75**	**0.84**	1.00	**<0.001**	**<0.001**
(95% CI)	(0.71 to 0.96)	(0.72 to 0.97)	(0.74 to 1.01)				(0.66 to 0.74)	(0.70 to 0.79)	(0.79 to 0.89)	(ref.)		
*p*-value vs. Q4	**0.012**	**0.020**	0.065	—	—	—	**<0.001**	**<0.001**	**<0.001**	—	—	—
Adjusted PR	**0.85**	**0.80**	**0.84**	1.00 (ref.)	**0.025**	**0.022**	**0.81**	**0.81**	**0.83**	1.00	**<0.001**	**<0.001**
(95% CI) ^‡^	(0.73 to 0.98)	(0.68 to 0.93)	(0.71 to <1.000)				(0.76 to 0.86)	(0.77 to 0.86)	(0.79 to 0.88)	(ref.)		
*p*-value vs. Q4	**0.029**	**0.004**	**0.045**	—	—	—	**<0.001**	**<0.001**	**<0.001**	—	—	—
Adjusted PD,	**−3.50**	**−4.59**	**−3.64**	0.00 (ref.)	—	—	**−5.48**	**−5.37**	**−4.83**	0.00	—	—
pp (95% CI) ^§^	(−6.63 to −0.38)	(−7.73 to −1.45)	(−7.20 to −0.09)				(−7.04 to −3.93)	(−6.88 to −3.86)	(−6.30 to −3.36)	(ref.)		
Healthy/traditional dietary pattern												
Crude PR	**1.84**	**1.50**	**1.28**	1.00 (ref.)	**<0.001**	**<0.001**	**1.41**	**1.20**	**1.09**	1.00	**<0.001**	**<0.001**
(95% CI)	(1.57 to 2.15)	(1.29 to 1.75)	(1.09 to 1.51)				(1.33 to 1.49)	(1.13 to 1.28)	(1.02 to 1.16)	(ref.)		
*p*-value vs. Q4	**<0.001**	**<0.001**	**0.003**	—	—	—	**<0.001**	**<0.001**	**0.008**	—	—	—
Adjusted PR	**1.75**	**1.37**	1.13	1.00 (ref.)	**<0.001**	**<0.001**	**1.34**	**1.17**	**1.09**	1.00	**<0.001**	**<0.001**
(95% CI) ^‡^	(1.45 to 2.10)	(1.16 to 1.61)	(0.95 to 1.34)				(1.26 to 1.42)	(1.10 to 1.24)	(1.02 to 1.17)	(ref.)		
*p*-value vs. Q4	**<0.001**	**<0.001**	0.162	—	—	—	**<0.001**	**<0.001**	**0.013**	—	—	—
Adjusted PD,	**11.27**	**5.55**	1.97	0.00 (ref.)	—	—	**7.44**	**3.66**	**1.96**	0.00	—	—
pp (95% CI) ^§^	(7.47 to 15.07)	(2.71 to 8.39)	(−0.79 to 4.74)				(5.96 to 8.92)	(2.20 to 5.13)	(0.41 to 3.50)	(ref.)		

Note: 95% CI, 95% confidence interval; PD, prevalence difference; PR, prevalence ratio; Q1–Q4, first through fourth adherence quartiles; ref., reference category. Q4 was the reference. Primary quartiles were defined separately within each survey edition and therefore represent relative positions rather than equivalent absolute cut points across 2015 and 2019. * Global *p*-values are design-adjusted Wald F tests jointly assessing Q1, Q2, and Q3 relative to Q4. ^†^ Trend *p*-values were obtained from quartiles coded 1–4. ^‡^ Adjusted for sex, race/skin color, school administrative sector, school location, geographic region, maternal educational attainment, household asset category, and co-residence with the mother and father. ^§^ Adjusted PDs were estimated by marginal standardization and are percentage-point differences relative to Q4. All tests were two-sided and used design-based *t* inference where applicable. Boldface indicates estimates with 95% CI excluding the null and *p*-values < 0.05.

**Table 5:** Adjusted continuous, ordinal, categorical, and between-survey comparisons of dietary-pattern associations with the survey-specific adverse mental health indicator among school-attending Brazilian adolescents aged 13–17 years.

Adjusted Analysis or Contrast	*PeNSE* 2015 Sample 2		*PeNSE* 2019		RPR, 2019 vs. 2015 (95% CI)	*p* for Heterogeneity
	PR	*p*-Value	PR	*p*-Value		
	(95% CI)		(95% CI)			
Ultra-processed/unhealthy dietary pattern						
Continuous score, per one *SD*	**1.08**	**0.010**	**1.09**	**<0.001**	1.01	0.756
	(1.02–1.14)		(1.06–1.11)		(0.95–1.07)	
Ordinal trend, per one-quartile increase	**1.06**	**0.022**	**1.07**	**<0.001**	1.01	0.726
	(1.01–1.12)		(1.05–1.09)		(0.96–1.07)	
Q1 vs. Q4	**0.85**	**0.029**	**0.81**	**<0.001**	0.96	0.612
	(0.73–0.98)		(0.76–0.86)		(0.82–1.13)	
Q2 vs. Q4	**0.80**	**0.004**	**0.81**	**<0.001**	1.02	0.803
	(0.68–0.93)		(0.77–0.86)		(0.87–1.20)	
Q3 vs. Q4	**0.84**	**0.045**	**0.83**	**<0.001**	0.99	0.937
	(0.71–1.00)		(0.79–0.88)		(0.83–1.19)	
Global quartile association/ between-survey heterogeneity	—	**0.025**	—	**<0.001**	*χ*^2^(3)	0.903
Healthy/traditional dietary pattern						
Continuous score, per one *SD*	**0.79**	**<0.001**	**0.89**	**<0.001**	**1.13**	**0.001**
	(0.74–0.85)		(0.87–0.91)		(1.05–1.21)	
Ordinal trend, per one-quartile increase	**0.83**	**<0.001**	**0.91**	**<0.001**	**1.10**	**0.004**
	(0.78–0.88)		(0.89–0.93)		(1.03–1.17)	
Q1 vs. Q4	**1.75**	**<0.001**	**1.34**	**<0.001**	**0.77**	**0.008**
	(1.45–2.10)		(1.26–1.42)		(0.63–0.93)	
Q2 vs. Q4	**1.37**	**<0.001**	**1.17**	**<0.001**	0.85	0.074
	(1.16–1.61)		(1.10–1.24)		(0.72–1.02)	
Q3 vs. Q4	1.13	0.162	**1.09**	**0.013**	0.96	0.694
	(0.95–1.34)		(1.02–1.17)		(0.80–1.16)	
Global quartile association/ between-survey heterogeneity	—	**<0.001**	—	**<0.001**	*χ*^2^(3)	**0.033**

Note: 95% CI, 95% confidence interval; PR, prevalence ratio; RPR, ratio of prevalence ratios; *SD*, standard deviation. RPRs use 2019 in the numerator; an RPR of 1 indicates no difference between editions. Survey-specific models used the same nine covariates as Table 4. Individual between-survey comparisons used Welch–Satterthwaite *t* inference combining survey-specific variances and design degrees of freedom. Global between-survey heterogeneity across Q1, Q2, and Q3 contrasts was assessed using a three-degree-of-freedom Wald chi-square test based on the complete covariance matrices. Boldface indicates estimates whose 95% CI excluding 1.00 and *p*-values < 0.05.

**Table 6:** Sensitivity analyses using the broader score-based adverse indicator and E-values among school-attending Brazilian adolescents aged 13–17 years.

Sensitivity Analysis or Contrast	2015 Result		2019 Result		RPR (95% CI)	*p* for Heterogeneity	ΔPR:
	PR	*p*-Value	PR	*p*-Value			2015
	(95% CI)		(95% CI)				2019
Ultra-processed/unhealthy dietary pattern							
Q1 vs. Q4	0.86	<0.001	0.83	<0.001	0.97	0.514	+0.014
	(0.79–0.94)		(0.80–0.87)		(0.88–1.07)		+0.021
Q2 vs. Q4	0.87	<0.001	0.87	<0.001	1.00	0.997	+0.069
	(0.80–0.94)		(0.84–0.89)		(0.92–1.09)		+0.052
Q3 vs. Q4	0.96	0.396	0.89	<0.001	0.93	0.180	+0.120
	(0.87–1.06)		(0.86–0.93)		(0.84–1.03)		+0.061
Continuous score,	1.07	<0.001	1.07	<0.001	1.00	0.935	−0.004
per one *SD*	(1.04–1.10)		(1.06–1.09)		(0.97–1.03)		−0.016
Ordinal trend,	1.06	<0.001	1.06	<0.001	1.00	0.810	−0.003
per one-quartile increase	(1.03–1.09)		(1.05–1.07)		(0.97–1.03)		−0.010
Global quartile association/	—	Global	—	Global	*χ*^2^(3)	0.557	—
between-survey heterogeneity		*p* < 0.001		*p* < 0.001			
E-value for primary	1.65; 1.15	—	1.77; 1.59	—	—	—	—
adjusted Q1 vs. Q4							
(point; CI limit)							
Healthy/traditional dietary pattern							
Q1 vs. Q4	1.45	<0.001	1.20	<0.001	**0.83**	**<0.001**	−0.295
	(1.33–1.59)		(1.16–1.25)		(0.75–0.91)		−0.137
Q2 vs. Q4	1.26	<0.001	1.10	<0.001	**0.88**	**0.005**	−0.110
	(1.16–1.37)		(1.06–1.15)		(0.80–0.96)		−0.064
Q3 vs. Q4	1.06	0.200	1.05	0.018	0.99	0.782	−0.068
	(0.97–1.17)		(1.01–1.09)		(0.89–1.09)		−0.042
Continuous score,	0.85	<0.001	0.93	<0.001	**1.09**	**<0.001**	+0.059
per one *SD*	(0.82–0.88)		(0.92–0.94)		(1.05–1.13)		+0.037
Ordinal trend,	0.88	<0.001	0.94	<0.001	**1.07**	**<0.001**	+0.051
per one-quartile increase	(0.85–0.90)		(0.93–0.95)		(1.04–1.11)		+0.032
Global quartile association/	—	Global	—	Global	*χ*^2^(3)	**<0.001**	—
between-survey heterogeneity		*p* < 0.001		*p* < 0.001			
E-value for primary	2.89; 2.26	—	2.02; 1.84	—	—	—	—
adjusted Q1 vs. Q4							
(point; CI limit)							

Note: 95% CI, 95% confidence interval; PR, prevalence ratio; RPR, ratio of prevalence ratios; *SD*, standard deviation; ΔPR, sensitivity estimate minus the corresponding primary unweighted-PCA estimate. Except for global test and E-value rows, the 2015 and 2019 result columns show the adjusted PR (95% CI) and *p*-value on separate lines. The alternative outcome included the upper-middle and highest adverse score categories. RPRs use 2019 in the numerator. Global between-survey heterogeneity was assessed using a three-degree-of-freedom Wald chi-square test. Boldface indicates significant between-survey heterogeneity (*p* < 0.05) and corresponding RPRs with 95% CI excluding 1.00. E-values represent the point estimate, and the confidence limit closest to the null for the primary adjusted Q1 vs. Q4 contrast. All ΔPR values were calculated from unrounded estimates.

**Table 7:** Robustness analyses based on survey-weighted PCA scores and weighted empirical quartiles among school-attending Brazilian adolescents aged 13–17 years.

Sensitivity Analysis or Contrast	2015 Adjusted Result		2019 Adjusted Result		RPR (95% CI)	*p* for Heterogeneity	ΔPR:
	PR	*p*-Value	PR	*p*-Value			2015
	(95% CI)		(95% CI)				2019
Ultra-processed/unhealthy dietary pattern							
Q1 vs. Q4	0.83	0.019	0.81	<0.001	0.97	0.703	−0.011
	(0.72–0.97)		(0.76–0.86)		(0.82–1.14)		−0.002
Q2 vs. Q4	0.80	0.005	0.82	<0.001	1.02	0.829	+0.002
	(0.68–0.94)		(0.77–0.87)		(0.86–1.20)		0.000
Q3 vs. Q4	0.85	0.052	0.83	<0.001	0.98	0.838	+0.006
	(0.71–1.00)		(0.79–0.88)		(0.82–1.17)		−0.004
Continuous score,	1.08	0.006	1.09	<0.001	1.01	0.755	+0.004
per one *SD*	(1.02–1.14)		(1.07–1.12)		(0.95–1.07)		+0.004
Ordinal trend,	1.07	0.016	1.07	<0.001	1.01	0.789	+0.004
per one-quartile increase	(1.01–1.12)		(1.05–1.10)		(0.95–1.06)		+0.002
Global quartile association/	—	Global	—	Global	*χ*^2^(3)	0.931	—
between-survey heterogeneity		*p* = 0.028		*p* < 0.001			
Healthy/traditional dietary pattern							
Q1 vs. Q4	1.77	<0.001	1.35	<0.001	**0.77**	**0.006**	+0.019
	(1.47–2.12)		(1.27–1.43)		(0.63–0.93)		+0.010
Q2 vs. Q4	1.38	<0.001	1.17	<0.001	0.85	0.058	+0.017
	(1.18–1.63)		(1.10–1.25)		(0.71–1.01)		+0.004
Q3 vs. Q4	1.12	0.200	1.09	0.013	0.97	0.775	−0.012
	(0.94–1.33)		(1.02–1.17)		(0.81–1.17)		0.000
Continuous score,	0.79	<0.001	0.89	<0.001	**1.12**	**0.002**	+0.001
per one *SD*	(0.74–0.85)		(0.87–0.91)		(1.05–1.21)		−0.001
Ordinal trend,	0.82	<0.001	0.91	<0.001	**1.10**	**0.003**	−0.004
per one-quartile increase	(0.77–0.87)		(0.89–0.92)		(1.03–1.18)		−0.003
Global quartile association/	—	Global	—	Global	*χ*^2^(3)	**0.023**	—
between-survey heterogeneity		*p* < 0.001		*p* < 0.001			

Note: 95% CI, 95% confidence interval; PCA, principal component analysis; PR, prevalence ratio; RPR, ratio of prevalence ratios; *SD*, standard deviation; ΔPR, weighted-PCA estimate minus the corresponding primary unweighted-PCA estimate. The 2015 and 2019 result columns show the adjusted PR (95% CI) and *p*-value on separate lines, except for global-test rows. Weighted PCA used final-weighted Pearson correlation matrices, two fixed components, Kaiser-normalized varimax rotation, and regression scoring. Weighted empirical quartiles were defined without interpolation and preserved ties. RPRs use 2019 in the numerator; global between-survey heterogeneity used a three-degree-of-freedom Wald chi-square test. Boldface indicates significant between-survey heterogeneity (*p* < 0.05) and corresponding RPR with 95% CI excluding 1.00. ΔPR values were calculated from unrounded estimates.

## Data Availability

The anonymized *PeNSE* 2015 and 2019 microdata, questionnaires, data dictionaries, and methodological documentation used in this study are publicly available on the *IBGE* official website: https://www.ibge.gov.br/estatisticas/sociais/saude/ (accessed on 23 June 2026). Supporting files, including the harmonized database, are available through the Open Science Framework (OSF™ Platform): https://doi.org/10.17605/OSF.IO/FUS4K (updated/accessed on 23 June 2026).

## Supplementary Materials

The following supporting information can be downloaded at: https://www.mdpi.com/article/10.3390/nu18172781/s1, STROBE Checklist S1: Strengthening the Reporting of Observational Studies in Epidemiology (STROBE) checklist for cross-sectional studies; Table S1: Construction of the survey-specific adverse mental health-related indicator scores and continuous-outcome sensitivity analyses; Table S2: Missing-data patterns, comparison of participants included in and excluded from the adjusted analyses, and inverse-probability-of-complete-case weighting sensitivity analyses.

## Abbreviations

The following abbreviations are used in this manuscript:

ASD	Absolute Standardized Difference
*CAAE*	*Certificado de Apresentação de Apreciação Ética* (Certificate of Presentation for Ethical Consideration)
*CD*	*Conselho Deliberativo* (Deliberative Council)
CI	Confidence Interval
*CONEP*	*Comissão Nacional de Ética em Pesquisa* (Brazilian National Research Ethics Commission)
*FAPES*	*Fundação de Amparo à Pesquisa do Espírito Santo* (Espírito Santo Research Support Foundation)
*FNDE*	*Fundo Nacional de Desenvolvimento da Educação* (Brazilian National Fund for Education Development)
*IBGE*	*Instituto Brasileiro de Geografia e Estatística* (Brazilian Institute of Geography and Statistics)
IPCCW	Inverse Probability of Complete Case Weighting
KMO	Kaiser–Meyer–Olkin
*LaDEEC*	*Laboratório de Delineamento de Estudos e Escrita Científica* (Laboratory for Study Design and Scientific Writing)
NR	Not Reported
OSF	Open Science Framework
PCA	Principal Component Analysis
PD	Prevalence Difference
*PeNSE*	*Pesquisa Nacional de Saúde do Escolar* (Brazilian National Survey of School Health)
*PNAE*	*Programa Nacional de Alimentação Escolar* (Brazilian National School Feeding Program)
pp	Percentage points
PR	Prevalence Ratio
Q1–Q4	First through fourth adherence quartiles
ROR	Research Organization Registry
RPR	Ratio of Prevalence Ratios
*SD*	Standard Deviation
*UFES*	*Universidade Federal do Espírito Santo*
ΔPR	Difference between a sensitivity or robustness PR estimate and the corresponding primary estimate
*h*^2^	Communality
*χ*^2^	Chi-square statistic

## References

[1] GBD 2019 Mental Disorders Collaborators (2022). Global, Regional, and National Burden of 12 Mental Disorders in 204 Countries and Territories, 1990–2019: A Systematic Analysis for the Global Burden of Disease Study 2019. Lancet Psychiatry.

[2] Patton G.C., Sawyer S.M., Santelli J.S., Ross D.A., Afifi R., Allen N.B., Arora M., Azzopardi P., Baldwin W., Bonell C. (2016). Our Future: A Lancet Commission on Adolescent Health and Wellbeing. Lancet.

[3] Solmi M., Radua J., Olivola M., Croce E., Soardo L., Salazar Pablo G., Il Shin J., Kirkbride J.B., Jones P., Kim J.H. (2022). Age at Onset of Mental Disorders Worldwide: Large-Scale Meta-Analysis of 192 Epidemiological Studies. Mol. Psychiatry.

[4] Caldwell D.M., Davies S.R., Hetrick S.E., Palmer J.C., Caro P., López-López J.A., Gunnell D., Kidger J., Thomas J., French C. (2019). School-Based Interventions to Prevent Anxiety and Depression in Children and Young People: A Systematic Review and Network Meta-Analysis. Lancet Psychiatry.

[5] Monteiro C.A., Cannon G., Levy R.B., Moubarac J.-C., Louzada M.L.C., Rauber F., Khandpur N., Cediel G., Neri D., Martinez-Steele E. (2019). Ultra-Processed Foods: What They Are and How to Identify Them. Public Health Nutr..

[6] Gonçalves H.V.B., Canella D.S., Bandoni D.H. (2020). Temporal Variation in Food Consumption of Brazilian Adolescents (2009–2015). PLoS ONE.

[7] Silva J.B., Elias B.C., Warkentin S., Mais L.A., Konstantyner T. (2022). Factors Associated with the Consumption of Ultra-Processed Food by Brazilian Adolescents: National Survey of School Health, 2015. Rev. Paul. Pediatr..

[8] Guerra P.H., Ribeiro E.H.C., Lopes R.F., Nunes L.M.B., Viali I.C., Ferraz B.P., Almeida I.A., Garzella M.H., Silveira J.A.C. (2023). Variables Associated with Ultra-Processed Foods Consumption among Brazilian Adolescents: A Systematic Review. Adolescents.

[9] Hu F.B. (2002). Dietary Pattern Analysis: A New Direction in Nutritional Epidemiology. Curr. Opin. Lipidol..

[10] Maugeri A., Barchitta M., Favara G., La Mastra C., La Rosa M.C., Magnano San Lio R., Agodi A. (2022). The Application of Clustering on Principal Components for Nutritional Epidemiology: A Workflow to Derive Dietary Patterns. Nutrients.

[11] Firth J., Gangwisch J.E., Borsini A., Wootton R.E., Mayer E.A. (2020). Food and Mood: How Do Diet and Nutrition Affect Mental Wellbeing?. BMJ.

[12] Marx W., Lane M., Hockey M., Aslam H., Berk M., Walder K., Borsini A., Firth J., Pariante C.M., Berding K. (2021). Diet and Depression: Exploring the Biological Mechanisms of Action. Mol. Psychiatry.

[13] Bogea E.G., Martins M.L.B., França A.K.T.C., Silva A.A.M. (2023). Dietary Patterns, Nutritional Status and Inflammatory Biomarkers in Adolescents from the RPS Birth Cohort Consortium. Nutrients.

[14] O’Neil A., Quirk S.E., Housden S., Brennan S.L., Williams L.J., Pasco J.A., Berk M., Jacka F.N. (2014). Relationship between Diet and Mental Health in Children and Adolescents: A Systematic Review. Am. J. Public Health.

[15] Lane M.M., Gamage E., Travica N., Dissanayaka T., Ashtree D.N., Gauci S., Lotfaliany M., O’Neil A., Jacka F.N., Marx W. (2022). Ultra-Processed Food Consumption and Mental Health: A Systematic Review and Meta-Analysis of Observational Studies. Nutrients.

[16] Tucker J., Brennan A., Benton D., Young H. (2025). A Recipe for Resilience: A Systematic Review of Diet and Adolescent Mental Health. Nutrients.

[17] Georgiou A., Chrysostomou S., Kantilafti M. (2026). Ultra-Processed Foods and Mental Health in Children and Adolescents: Evidence from a Systematic Review. Nutrients.

[18] Gratão L.H.A., Silva T.P.R., Rocha L.L., Jardim M.Z., Oliveira T.R.P.R., Cunha C.F., Mendes L.L. (2024). Common Mental Disorders in Brazilian Adolescents: Association with School Characteristics, Consumption of Ultra-Processed Foods and Waist-to-Height Ratio. Cad. Saude Publica.

[19] Faisal-Cury A., Leite M.A., Escuder M.M.L., Levy R.B., Peres M.F.T. (2022). The Relationship between Ultra-Processed Food Consumption and Internalising Symptoms among Adolescents from São Paulo City, Southeast Brazil. Public Health Nutr..

[20] Mesas A.E., González A.D., Andrade S.M., Martínez-Vizcaíno V., López-Gil J.F., Jiménez-López E. (2022). Increased Consumption of Ultra-Processed Food Is Associated with Poor Mental Health in a Nationally Representative Sample of Adolescent Students in Brazil. Nutrients.

[21] de Oliveira I.F.R., Pereira N.G., Monteiro L.F., de Rezende L.M.T., de Lira C.A.B., Monfort-Pañego M., da Costa W.P., Noll P.R.E.S., Noll M. (2025). Factors Influencing the Quality of Life and Mental Health of Brazilian Federal Education Network Employees: An Epidemiological Cross-Sectional Study. Heliyon.

[22] IBGE (2016). Pesquisa Nacional de Saúde do Escolar: 2015.

[23] IBGE (2021). Pesquisa Nacional de Saúde do Escolar: 2019.

[24] Vale D., Lyra C., Dantas N., Andrade M., Oliveira A. (2022). Dietary and Nutritional Profiles among Brazilian Adolescents. Nutrients.

[25] Haddad M.R., Sarti F.M. (2024). Determinants of Inequalities in the Exposure to and Adoption of Multiple Health Risk Behaviors among Brazilian Adolescents, 2009–2019. Eur. J. Investig. Health Psychol. Educ..

[26] Ferreira A.C.M., Silva A.G., Morais É.A.H., Malta D.C. (2024). National School Health Survey: Methodological Aspects Changes and Comparability with the Global School-Based Student Health Survey. Rev. Bras. Epidemiol..

[27] Levin K.A. (2006). Study Design III: Cross-Sectional Studies. Evid. Based. Dent..

[28] Mann C.J. (2003). Observational Research Methods. Research Design II: Cohort, Cross Sectional, and Case-Control Studies. Emerg. Med. J..

[29] Abreu L.C. (2026). Observational Studies in Health: Epidemiological Designs, Analytical Potential, and Limits in Causal Inference. J. Hum. Growth Dev..

[30] IBGE (2015). PeNSE—Pesquisa Nacional de Saúde do Escolar: Edição. https://www.ibge.gov.br/estatisticas/sociais/saude/9134-pesquisa-nacional-de-saude-do-escolar.html?edicao=9135.

[31] IBGE (2019). PeNSE—Pesquisa Nacional de Saúde do Escolar: Edição. https://www.ibge.gov.br/estatisticas/sociais/saude/9134-pesquisa-nacional-de-saude-do-escolar.html?edicao=31442.

[32] Cattell R.B. (1966). The Scree Test for the Number of Factors. Multivar. Behav. Res..

[33] Kaiser H.F. (1960). The Application of Electronic Computers to Factor Analysis. Educ. Psychol. Meas..

[34] Horn J.L. (1965). A Rationale and Test for the Number of Factors in Factor Analysis. Psychometrika.

[35] Kaiser H.F., Rice J. (1974). Little Jiffy, Mark IV. Educ. Psychol. Meas..

[36] Bartlett M.S. (1950). Tests of Significance in Factor Analysis. Br. J. Stat. Psychol..

[37] Kaiser H.F. (1958). The Varimax Criterion for Analytic Rotation in Factor Analysis. Psychometrika.

[38] Cronbach L.J. (1951). Coefficient Alpha and the Internal Structure of Tests. Psychometrika.

[39] Carvajal-Velez L., Ahs J.W., Requejo J.H., Kieling C., Lundin A., Kumar M., Luitel N.P., Marlow M., Skeen S., Tomlinson M. (2023). Measurement of Mental Health among Adolescents at the Population Level: A Multicountry Protocol for Adaptation and Validation of Mental Health Measures. J. Adolesc. Health.

[40] Uzêda J.C.O., Ribeiro-Silva R.C., Silva N.J., Fiaccone R.L., Malta D.C., Ortelan N., Barreto M.L. (2019). Factors Associated with the Double Burden of Malnutrition among Adolescents, National Adolescent School-Based Health Survey (PeNSE 2009 and 2015). PLoS ONE.

[41] Howe L.D., Hargreaves J.R., Huttly S.R.A. (2008). Issues in the Construction of Wealth Indices for the Measurement of Socio-Economic Position in Low-Income Countries. Emerg. Themes Epidemiol..

[42] Filmer D., Pritchett L.H. (2001). Estimating Wealth Effects without Expenditure Data—Or Tears: An Application to Educational Enrollments in States of India. Demography.

[43] Lumley T. (2004). Analysis of Complex Survey Samples. J. Stat. Softw..

[44] Lumley T., Scott A. (2014). Tests for Regression Models Fitted to Survey Data. Aust. N. Z. J. Stat..

[45] Barros A.J.D., Hirakata V.N. (2003). Alternatives for Logistic Regression in Cross-Sectional Studies: An Empirical Comparison of Models That Directly Estimate the Prevalence Ratio. BMC Med. Res. Methodol..

[46] Zou G. (2004). A Modified Poisson Regression Approach to Prospective Studies with Binary Data. Am. J. Epidemiol..

[47] Bieler G.S., Brown G.G., Williams R.L., Brogan D.J. (2010). Estimating Model-Adjusted Risks, Risk Differences, and Risk Ratios from Complex Survey Data. Am. J. Epidemiol..

[48] Altman D.G., Bland J.M. (2003). Interaction Revisited: The Difference between Two Estimates. BMJ.

[49] Satterthwaite F.E. (1946). An Approximate Distribution of Estimates of Variance Components. Biom. Bull..

[50] Welch B.L. (1947). The Generalization of “Student’s” Problem When Several Different Population Variances Are Involved. Biometrika.

[51] Lorenzo-Seva U., ten Berge J.M.F. (2006). Tucker’s Congruence Coefficient as a Meaningful Index of Factor Similarity. Methodology.

[52] Seaman S.R., White I.R. (2013). Review of Inverse Probability Weighting for Dealing with Missing Data. Stat. Methods Med. Res..

[53] VanderWeele T.J., Ding P. (2017). Sensitivity Analysis in Observational Research: Introducing the E-Value. Ann. Intern. Med..

[54] Wang D., Li Y., Wang X., Liu X., Fu B., Lin Y., Larsen L., Offen W. (2015). Overview of Multiple Testing Methodology and Recent Development in Clinical Trials. Contemp. Clin. Trials.

[55] R Core Team (2025). R: A Language and Environment for Statistical Computing.

[56] Lumley T., Gao P., Schneider B., Kolenkikov S. (2026). Survey: Analysis of Complex Survey Samples.

[57] Vandenbroucke J.P., von Elm E., Altman D.G., Gøtzsche P.C., Mulrow C.D., Pocock S.J., Poole C., Schlesselman J.J., Egger M. (2007). Strengthening the Reporting of Observational Studies in Epidemiology (STROBE). Epidemiology.

[58] Kazmierski K.F.M., Borelli J.L., Rao U. (2022). Negative Affect, Childhood Adversity, and Adolescents’ Eating Following Stress. Appetite.

[59] Schneider-Worthington C.R., Smith K.E., Roemmich J.N., Salvy S.-J. (2022). External Food Cue Responsiveness and Emotional Eating in Adolescents: A Multimethod Study. Appetite.

[60] Gomes D.R., Santos-Neto E.T., Salaroli L.B. (2025). Adverse Childhood Experiences and Their Implications for the Prevalence of Overweight Adolescents in One Metropolitan Region of Brazil. J. Hum. Growth Dev..

[61] Alves S.A.A., Bezerra I.M.P., Albuquerque G.A., Cavalcante E.G.R., Lopes M.S.V. (2021). Sustainable Practices as Actions to Promote Adolescent Health. J. Hum. Growth Dev..

[62] Brazil Resolution CD/FNDE No. 4 of February 26, 2026: Regulations on the Management and Provision of School Meals to Basic Education Students Under the National School Feeding Program (PNAE). https://www.gov.br/fnde/pt-br/acesso-a-informacao/legislacao/resolucoes/2026/resolucao-cd_fnde-no-4-de-26-de-fevereiro-de-2026.pdf.

[63] Cattafesta M., Salaroli L.B. (2024). Beyond Ultra-Processed Foods: The New Direction of the Basic Food Basket in Brazil. J. Hum. Growth Dev..

[64] Samad N., Bearne L., Noor F.M., Akter F., Parmar D. (2024). School-Based Healthy Eating Interventions for Adolescents Aged 10–19 Years: An Umbrella Review. Int. J. Behav. Nutr. Phys. Act..

