# Supplementary material for: Dietary Patterns and an Adverse Mental Health Indicator Among School-Attending Brazilian Adolescents: Analyses of the 2015 and 2019 PeNSE Surveys
Source: Nutrients. 2026 Aug 25;18(17):2781. doi: 10.3390/nu18172781 (PMC13567332; doi:10.3390/nu18172781)
Supplement: Supplementary file 1 [file nutrients-18-02781-s001.zip › nutrients-4459206-supplementary.pdf]

Table S1: Construction of the survey-specific adverse mental health-related indicator scores and continuous-outcome sensitivity analyses.

Panel A. Adverse mental health-related indicator score composition and adverse-oriented coding.

Edition	Item / source variable	Question content and recall period	Original response structure	Contribution to adverse-oriented score
2015	Parental understanding<br>Source: pais_entendem	Past 30 days: frequency with which parents/guardians understood the student's problems and worries.	Never, rarely, sometimes, most of the time, always.	Reverse-coded: 5, 4, 3, 2, 1.
	Peer support<br>Source: colegas_tratam_bem	Past 30 days: frequency with which school peers treated the student well and/or were helpful.	Never, rarely, sometimes, most of the time, always.	Reverse-coded: 5, 4, 3, 2, 1.
	Peer mistreatment<br>Source: colegas_esculachou	Past 30 days: frequency of being mocked, intimidated, or humiliated by school peers to the point of feeling hurt or offended.	Never, rarely, sometimes, most of the time, always.	Retained in adverse direction: 1, 2, 3, 4, 5.
	Experienced bullying<br>Source: sofreu_bullying	Lifetime report of having experienced bullying.	Yes or no.	Yes = 2; no = 1.
	Loneliness<br>Source: sente_sozinho	Past 12 months: frequency of feeling lonely.	Never, rarely, sometimes, most of the time, always.	Retained in adverse direction: 1, 2, 3, 4, 5.
	Sleep loss due to worry<br>Source: nao_dormir_preocupado	Past 12 months: frequency of being unable to sleep at night because of being very worried.	Never, rarely, sometimes, most of the time, always.	Retained in adverse direction: 1, 2, 3, 4, 5.
	Close friends<br>Source: quantos_amigos_proximos	Number of close friends.	None, one, two, three or more.	None = 4; one = 3; two = 2; three or more = 1.
	Total score and categories	Sum of the seven nonmissing item scores.	Theoretical range: 7–31.	Weighted distribution categories: ≤11, 12–14, 15–17, ≥18. Primary adverse indicator: ≥18. Broader sensitivity indicator: ≥15 vs. ≤14.
2019	Close friends<br>Source: amigos_proximos	Number of close friends.	Three or more, two, one, none.	Three or more = 0; two = 1; one = 2; none = 3.
	Worry<br>Source: preocupacao_30dias	Past 30 days: frequency of feeling very worried about ordinary daily matters.	Never, rarely, sometimes, most of the time, always.	Retained in adverse direction: 1, 2, 3, 4, 5.
	Sadness<br>Source: tristeza_30dias	Past 30 days: frequency of feeling sad.	Never, rarely, sometimes, most of the time, always.	Retained in adverse direction: 1, 2, 3, 4, 5.
	Perceived lack of care<br>Source: ning_preocupa_30dias	Past 30 days: frequency of feeling that nobody cared about the student.	Never, rarely, sometimes, most of the time, always.	Retained in adverse direction: 1, 2, 3, 4, 5.
	Irritability/nervousness<br>Source: irritado_nervoso_30dias	Past 30 days: frequency of feeling irritated, nervous, or in a bad mood.	Never, rarely, sometimes, most of the time, always.	Retained in adverse direction: 1, 2, 3, 4, 5.
	Life not worth living<br>Source: vida_sem_sentido_30dias	Past 30 days: frequency of feeling that life was not worth living.	Never, rarely, sometimes, most of the time, always.	Retained in adverse direction: 1, 2, 3, 4, 5.
	Total score and categories	Sum of the six nonmissing item scores.	Theoretical range: 5–28.	Weighted distribution categories: ≤11, 12–15, 16–18, ≥19. Primary adverse indicator: ≥19. Broader sensitivity indicator: ≥16 vs. ≤15.

Note: Question wording is presented as a concise English translation of the *PeNSE* questionnaires. Higher item and total scores indicate a greater burden of the adverse mental health-related experiences and symptoms represented in the corresponding survey-specific adverse mental health-related indicator score.

Panel B. Continuous standardized-outcome sensitivity analysis.

Dietary pattern	Exposure contrast	2015 beta (95% CI)	*p*-value	2019 beta (95% CI)	*p*-value
Ultra-processed/unhealthy	Q1 vs. Q4	–0.169 (–0.250 to –0.087)	<0.001	–0.199 (-0.236 to –0.162)	<0.001
	Q2 vs. Q4	–0.137 (–0.210 to –0.065)	<0.001	–0.152 (-0.185 to –0.120)	<0.001
	Q3 vs. Q4	–0.054 (–0.132 to 0.025)	0.178	–0.125 (-0.159 to –0.092)	<0.001
	Per 1-*SD* increase	0.074 (0.045 to 0.103)	<0.001	0.075 (0.062 to 0.088)	<0.001
Healthy/traditional	Q1 vs. Q4	0.400 (0.313 to 0.487)	<0.001	0.218 (0.183 to 0.253)	<0.001
	Q2 vs. Q4	0.245 (0.174 to 0.317)	<0.001	0.111 (0.076 to 0.146)	<0.001
	Q3 vs. Q4	0.097 (0.021 to 0.173)	0.012	0.063 (0.030 to 0.097)	<0.001
	Per 1-*SD* increase	–0.162 (–0.195 to –0.129)	<0.001	–0.081 (–0.093 to –0.068)	<0.001

Note: Panel B reports survey-weighted adjusted mean differences in the standardized survey-specific adverse mental health-related indicator score. Coefficients are interpretable within surveys only and do not establish psychometric validity or measurement equivalence between editions. Items were summed without differential weighting and without imputation; complete item data were required. These scores were designed for survey-specific epidemiological ranking and population surveillance. They are not validated diagnostic scales, and their raw scores, categories, and thresholds are not metrically equivalent between 2015 and 2019.

Table S2: Missing-data patterns, comparison of participants included in and excluded from the adjusted analyses, and inverse-probability-of-complete-case weighting sensitivity analyses.

Panel A. Missingness in PCA-eligible samples, by survey edition.

Variable	2015			2019		
	**Missing *n***	**Unweighted %**	**Weighted %**	**Missing *n***	**Unweighted %**	**Weighted %**
Primary adverse-score indicator	291	2.7	2.9	1,176	0.9	1.1
Sex	0	0.0	0.0	288	0.2	0.1
Race/skin color	7	0.1	0.1	2,488	2.0	0.8
School administrative sector	0	0.0	0.0	0	0.0	0.0
School location	0	0.0	0.0	0	0.0	0.0
Geographic region	0	0.0	0.0	0	0.0	0.0
Maternal educational attainment	2,335	21.6	23.5	19,593	15.8	20.7
Household asset category	31	0.3	0.4	205	0.2	0.2
Co-residence with mother	2	0.0	0.0	32	0.0	0.0
Co-residence with father	2	0.0	0.0	73	0.1	0.1
Excluded from adjusted model (any required outcome/covariate missing)	2,536	23.4	25.5	22,841	18.4	22.3

Notes: Percentages use the survey-specific PCA-eligible sample as the denominator (n = 10,835 in 2015 and n = 124,194 in 2019). Weighted percentages use the final sampling weights. The last row represents exclusion for any missing primary outcome or prespecified adjustment variable and therefore is not the sum of the variable-specific rows because missingness can overlap.

Panel B. Weighted comparison of participants included in and excluded from adjusted analyses.

Characteristic	Category/statistic	2015			2019		
		Included	Excluded	ASD	Included	Excluded	ASD
Sex	Male	49.1%	53.5%	0.089	47.4%	55.3%	0.159
	Female	50.9%	46.5%	0.089	52.6%	44.4%	0.165
	Missing/not reported	—	—	—	0.0%	0.3%	0.077
Race/skin color	White	37.8%	31.9%	0.126	37.3%	30.5%	0.145
	Black	12.2%	15.9%	0.104	13.0%	14.9%	0.054
	Asian	3.8%	5.0%	0.060	3.7%	3.8%	0.005
	Brown/mixed	43.5%	43.5%	0.001	43.0%	43.8%	0.016
	Indigenous	2.6%	3.5%	0.048	3.0%	3.6%	0.036
	Missing/not reported	0.0%	0.3%	0.071	0.0%	3.4%	0.267
Geographic region	North	9.4%	8.9%	0.016	10.8%	10.9%	0.002
	Northeast	27.7%	30.3%	0.057	28.6%	27.7%	0.019
	Southeast	41.1%	41.3%	0.004	38.2%	40.5%	0.046
	South	13.8%	12.9%	0.026	13.9%	13.4%	0.015
	Central-West	8.0%	6.6%	0.055	8.5%	7.5%	0.036
School administrative sector	Public	84.8%	93.5%	0.282	83.6%	92.2%	0.266
	Private	15.2%	6.5%	0.282	16.4%	7.8%	0.266
School location	Urban	95.4%	92.0%	0.141	92.8%	91.0%	0.069
	Rural	4.6%	8.0%	0.141	7.2%	9.0%	0.069

Characteristic	Category/statistic	2015			2019		
		Included	Excluded	ASD	Included	Excluded	ASD
Maternal educational attainment	0–8 years	32.0%	4.1%	0.778	36.8%	3.0%	0.935
	9–11 years	43.6%	2.3%	1.126	34.3%	2.0%	0.922
	≥12 years	24.4%	1.3%	0.734	29.0%	1.9%	0.807
	Missing/not reported	0.0%	92.2%	4.871	0.0%	93.1%	5.197
Household asset category	Lower asset-score category	15.7%	26.0%	0.256	21.4%	29.0%	0.174
	Intermediate asset-score category	53.0%	52.6%	0.008	60.8%	59.4%	0.029
	Higher asset-score category	31.3%	19.9%	0.263	17.8%	10.6%	0.208
	Missing/not reported	0.0%	1.5%	0.173	0.0%	1.1%	0.146
Co-residence with mother	Yes	90.0%	83.9%	0.180	89.4%	83.4%	0.177
	No	10.0%	16.0%	0.179	10.6%	16.5%	0.174
	Missing/not reported	0.0%	0.1%	0.036	0.0%	0.1%	0.044
Co-residence with father	Yes	62.8%	58.1%	0.096	60.8%	59.0%	0.037
	No	37.2%	41.8%	0.093	39.2%	40.7%	0.030
	Missing/not reported	0.0%	0.1%	0.046	0.0%	0.3%	0.078
Ultra-processed/unhealthy pattern quartile	Q1	24.1%	27.1%	0.069	25.8%	32.7%	0.153
	Q2	24.7%	25.3%	0.013	25.0%	24.2%	0.019
	Q3	25.8%	23.8%	0.047	24.2%	21.5%	0.065
	Q4	25.4%	23.8%	0.037	25.0%	21.6%	0.080

Characteristic	Category/statistic	2015			2019		
		Included	Excluded	ASD	Included	Excluded	ASD
Healthy/traditional pattern quartile	Q1	24.0%	28.7%	0.107	23.9%	28.2%	0.097
	Q2	25.5%	25.2%	0.007	26.1%	26.0%	0.003
	Q3	25.4%	24.6%	0.017	25.3%	23.6%	0.041
	Q4	25.1%	21.4%	0.087	24.7%	22.3%	0.056
Primary adverse-score indicator	No	80.2%	72.0%	0.195	74.9%	74.7%	0.004
	Yes	19.8%	16.7%	0.081	25.1%	20.1%	0.119
	Missing/not reported	0.0%	11.4%	0.507	0.0%	5.2%	0.330
Ultra-processed/unhealthy component score	Mean (weighted *SD*)	0.03 (1.00)	–0.05 (1.02)	0.081	–0.01 (1.01)	–0.13 (1.05)	0.115
Healthy/traditional component score	Mean (weighted *SD*)	0.02 (0.98)	–0.09 (1.01)	0.112	0.02 (0.98)	–0.07 (1.00)	0.084
Adverse mental health-related indicator total score	Mean (weighted *SD*)	14.21 (3.89)	14.02 (3.94)	0.047	15.15 (4.75)	14.30 (4.89)	0.177

Panel C. Focal Q1 vs. Q4 complete-case-weighted prevalence-ratio estimates.

**Survey**	**Dietary pattern**	**Primary PR (95% CI)**	**IPCCW PR (95% CI)**	**IPCCW *p*-value**	**∣ΔPR∣**
2015	Ultra-processed/unhealthy	0.85 (0.73–0.98)	0.84 (0.71–0.98)	0.024	0.015
	Healthy/traditional	1.75 (1.45–2.10)	1.75 (1.45–2.11)	<0.001	0.001
2019	Ultra-processed/unhealthy	0.81 (0.76–0.86)	0.80 (0.75–0.85)	<0.001	0.013
	Healthy/traditional	1.34 (1.26–1.42)	1.33 (1.25–1.42)	<0.001	0.008

Panel D. Complete-case propensity and stabilized-weight diagnostics.

**Survey**	**Eligible *n***	**Complete-case *n***	**Weighted P(complete)**	**Predicted probability range**	**Stabilized factor median**	**Stabilized factor range**
2015	10,544	8,299	0.767	0.602–0.933	0.981	0.822–1.269
2019	123,018	101,353	0.786	0.582–0.940	0.927	0.837–1.352

Notes: IPCCW, inverse-probability-of-complete-case weighting. These analyses assess robustness to the modeled complete-case selection mechanism but cannot prove missing at random or exclude nonignorable selection bias. ASD, absolute standardized difference. Values are sampling-weighted percentages unless shown as mean (weighted population *SD*). The comparison used the PCA-eligible samples; "included" denotes participants retained in the adjusted analytical domain, and "excluded" denotes those omitted because the primary outcome or at least one prespecified covariate was missing. Missing or unreported values were retained as explicit categories for categorical comparisons. ASDs are descriptive measures of imbalance and were not used as hypothesis tests.

STROBE Checklist S1: Strengthening the Reporting of Observational Studies in Epidemiology (STROBE) checklist for cross-sectional studies

Section	Item No.	Recommendation	Location in the manuscript
**Title and abstract**	1(a)	Indicate the study's design with a commonly used term in the title or the abstract	Abstract: identifies two independent cross-sectional surveys; Methods, Section 2.1.
	1(b)	Provide in the abstract an informative and balanced summary of what was done and what was found	Abstract: Background/Objectives, Methods, Results, and cautious Conclusions.
**Introduction**			
Background/ rationale	2	Explain the scientific background and rationale for the investigation being reported	Introduction, paragraphs 1–6.
Objectives	3	State specific objectives, including any prespecified hypotheses	End of Introduction: study objective and analytical purpose.
**Methods**			
Study design	4	Present key elements of study design early in the paper	Section 2.1: secondary analysis of two independent cross-sectional *PeNSE* editions.
Setting	5	Describe the setting, locations, and relevant dates, including periods of recruitment, exposure, follow-up, and data collection	Sections 2.1–2.2: nationwide Brazilian school-based surveys, 2015 and 2019, public and private schools.
Participants	6(a)	Give the eligibility criteria, and the sources and methods of selection of participants	Section 2.2 and Table 1: age eligibility, Sample 2 restriction in 2015, dietary completeness, outcome/design/covariate requirements.
Variables	7	Clearly define all outcomes, exposures, predictors, potential confounders, and effect modifiers. Give diagnostic criteria, if applicable	Sections 2.3–2.6; the outcome is defined as the survey-specific adverse mental health indicator derived from a survey-specific adverse mental health-related indicator score; Supplementary Table S1 documents item composition and coding.
Data sources/ measurement	8*	For each variable of interest, give sources of data and details of methods of assessment (measurement). Describe comparability of assessment methods if there is more than one group	Sections 2.1–2.6; between-edition measurement differences are explicitly described; Supplementary Table S1 documents source items, adverse-oriented coding, and construction of the survey-specific adverse mental health-related indicator score.
Bias	9	Describe any efforts to address potential sources of bias	Sections 2.6 and 2.7.4; complete-case comparison, IPCCW, E-values, weighted PCA, age-group and continuous-outcome sensitivity analyses; Section 4.4.
Study size	10	Explain how the study size was arrived at	Section 2.2 and Table 1: all eligible records in the public-use survey files; no separate sample-size calculation for this secondary analysis.
Quantitative variables	11	Explain how quantitative variables were handled in the analyses. If applicable, describe which groupings were chosen and why	Sections 2.3–2.7: 0–7-day dietary markers, PCA scores, standardization, empirical quartiles, survey-specific adverse mental health-related indicator score thresholds, asset categories, and age-group sensitivity.

Section	Item No.	Recommendation	Location in the manuscript
Statistical methods	12(a)	Describe all statistical methods, including those used to control for confounding	Sections 2.7.1–2.7.4: survey-weighted modified Poisson models, prespecified covariate set, marginal standardization, continuous and ordinal models.
	12(b)	Describe any methods used to examine subgroups and interactions	Section 2.7.3: between-survey RPR and global heterogeneity tests; no pooled exposure-by-year interaction.
	12(c)	Explain how missing data were addressed	Sections 2.2, 2.6, and 2.7.4; complete-case primary analyses, missingness description, included/excluded comparison, and IPCCW sensitivity.
	12(d)	If applicable, describe analytical methods taking account of sampling strategy	Section 2.7: weights, strata, PSUs, Taylor linearization, domain analysis, singleton-PSU handling, and design degrees of freedom.
	12(e)	Describe any sensitivity analyses	Section 2.7.4; Table S1, Figure 1, and Table S2.
**Results**			
Participants	13(a)*	Report numbers of individuals at each stage of study—e.g. numbers potentially eligible, examined for eligibility, confirmed eligible, included in the study, completing follow-up, and analyzed	Section 3.1 and Table 1.
	13(b)*	Give reasons for non-participation at each stage	Table 1 note and Table S2, Panel A: missing outcome/covariate information is reported by variable; maternal education was the main source of adjusted-sample loss.
	13(c)*	Consider use of a flow diagram	Table 1 provides a complete numerical selection sequence; a separate flow diagram was not required because stages are directly tabulated.
Descriptive data	14(a)*	Give characteristics of study participants (e.g. demographic, clinical, social) and information on exposures and potential confounders	Section 3.2 and Table 2.
	14(b)*	Indicate number of participants with missing data for each variable of interest	Table S2, Panel A.
Outcome data	15*	Report numbers of outcome events or summary measures	Section 3.3: weighted prevalence of membership in the primary survey-specific adverse mental health indicator category; Table S1, Panel B, reports continuous-outcome sensitivity results.
Main results	16(a)	Give unadjusted estimates and, if applicable, confounder-adjusted estimates and their precision (e.g., 95% confidence interval). Make clear which confounders were adjusted for and why they were included	Table 4 and its note; Sections 2.6 and 3.5; 95% confidence intervals reported.
	16(b)	Report category boundaries when continuous variables were categorized	Sections 2.4–2.5; survey-specific adverse mental health-related indicator score categories and dietary-pattern quartile definitions; Table S1.
	16(c)	If relevant, consider translating estimates of relative risk into absolute risk for a meaningful time period	Table 4 and Figure 2: adjusted prevalence differences in percentage points.

Section	Item No.	Recommendation	Location in the manuscript
Other analyses	17	Report other analyses done—e.g. analyses of subgroups and interactions, and sensitivity analyses	Sections 3.6–3.7; Tables 5–7; Table S1, Figure 1, and Table S2.
**Discussion**			
Key results	18	Summarize key results with reference to study objectives	Section 4.1.
Limitations	19	Discuss limitations of the study, taking into account sources of potential bias or imprecision. Discuss both direction and magnitude of any potential bias	Section 4.4: cross-sectional temporality, measurement limitations, marginal KMO, complete-case selection, residual confounding, comparability, multiplicity, and generalizability.
Interpretation	20	Give a cautious overall interpretation of results considering objectives, limitations, multiplicity of analyses, results from similar studies, and other relevant evidence	Sections 4.1–4.3 and Conclusion; causal and temporal-change claims explicitly avoided.
Generalizability	21	Discuss the generalizability (external validity) of the study results	Section 4.4: findings apply to school-attending adolescents represented by the survey design and should not be generalized to adolescents outside the school-attending population.
**Other information**			
Funding	22	Give the source of funding and the role of the funders for the present study and, if applicable, for the original study on which the present article is based	Funding statement: no external funding; institutional acknowledgments reported separately.

Note: This checklist is a reporting aid and not an instrument for judging methodological quality. An Explanation and Elaboration article discusses each checklist item and gives methodological background and published examples of transparent reporting. Information on the STROBE Initiative is available at www.strobe-statement.org. *Give information separately for exposed and unexposed groups.
